# Real-World Health Care Resource Utilization and Economic Burden Among Patients with Idiopathic Pulmonary Fibrosis in Commercially Insured and Medicare Advantage Populations in the United States

**DOI:** 10.36469/001c.161503

**Published:** 2026-06-03

**Authors:** Joseph Yang, Rebecca Fee, Andrea Steffens, Lisa Le, Bonnie H. Bui, Bijan J. Borah

**Affiliations:** 1 Boehringer Ingelheim Pharmaceuticals Inc. Ridgefield, Connecticut; 2 Optum, Eden Prairie, Minnesota; 3 College of Medicine and Science Mayo Clinic, Rochester, Minnesota

**Keywords:** Idiopathic pulmonary fibrosis, costs, antifibrotic treatments, healthcare resource utilization

## Abstract

**Background:** The economic burden associated with idiopathic pulmonary fibrosis (IPF) is not well understood, especially since the approval of antifibrotic medications to treat IPF in 2014. **Objective:** This study assessed healthcare resource utilization (HCRU) and costs for patients with and without IPF among insured patients in the United States. **Methods:** This retrospective study used claims data from the Optum Research Database from 01 January 2016 through 31 December 2023. The IPF cohort included patients with at least 2 claims for IPF within 365 days. A comparator cohort was exact matched to the IPF cohort in a 4:1 ratio on demographic characteristics. Follow-up all-cause and respiratory-related HCRU and costs were collected. Outcomes were presented as weighted per-patient per-month (wPPPM) to account for variable follow-up. Descriptive results were reported by cohort, overall, and stratified by insurance type. All-cause and respiratory-related wPPPM costs were modeled using generalized linear models and time to all-cause and respiratory-related inpatient visits were modeled using Cox proportional hazards regression models, adjusted for baseline comorbidities. **Results:** A total of 7582 patients were included in the IPF cohort; 10.4% were commercially insured and 89.6% insured by Medicare Advantage Prescription Drug (MAPD) plans. The comparator cohort included 30 328 demographically matched controls. Overall, mean all-cause wPPPM counts of HCRU during follow-up were significantly higher in the IPF cohort than the comparator cohort for all categories (all P < .001). Similarly, mean (SD) follow-up all-cause wPPPM total healthcare costs were higher for the IPF cohort than the comparator cohort (4667[8252] vs 1196[2114], P < .001). After adjustment, the cost ratio for all-cause total healthcare costs between the IPF and comparator cohorts was 3.4 (95% confidence interval [CI], 3.10-3.64), and patients in the IPF cohort were 2.1 times (95% CI, 1.97-2.15) more likely to be hospitalized for all causes than the comparator cohort. For both HCRU and costs, similar patterns were observed in both the commercial and MAPD populations. Conclusion: Patients with IPF incurred substantially higher HCRU and costs than the demographically matched comparator cohort. Study results suggest the need for treatment options to improve management of IPF and reduce costly HCRU.

## INTRODUCTION

Idiopathic pulmonary fibrosis (IPF) is a type of interstitial lung disease (ILD) characterized by chronic, progressive scarring of the lungs which makes breathing increasingly difficult.^1^ The adjusted incidence rate and prevalence of IPF in the US between 2017 and 2022 were 9.8 to 18.4 cases per 100 000 person-years and 34.4 to 67.1 cases per 100 000 persons.^2^ IPF is incurable and irreversible, with the median survival following diagnosis being approximately 3 to 5 years.^1,3^

Although there is no curative treatment for IPF currently available, two antifibrotic medications, nintedanib and pirfenidone, were approved by the US Food and Drug Administration in 2014.^4–8^ Antifibrotic treatments slow the rate of lung function decline, aiming to mitigate deterioration in quality of life. Antifibrotics have become the mainstay of IPF treatment and are currently recommended by the American Thoracic Society (ATS) for the treatment of patients with IPF.^1^ However, gastrointestinal side effects associated with antifibrotics have been identified as a major barrier to their consistent use.^9^

Previous studies have shown that IPF is associated with significant economic burden to patients and the healthcare system.^10–12^ A systematic review suggested that the estimated annual per-patient cost of IPF was approximately $20 000 in the US, approximately 2.5 to 3.5 times higher than the national healthcare expenditure.^13^ Collard et al reported the healthcare resource utilization (HCRU) in patients with IPF compared with their matched controls using 2 claims databases.^14,15^ Using the Medicare fee-for-service data, they found that patients with IPF had a 1.34-fold higher risk of hospitalization and emergency room (ER) visits, and higher total annual medical costs ($20 887 vs $8932), compared with the control group.^15^ Similarly, when using the commercial claims data, the risk of all-cause admission and outpatient visits was around 2-fold higher in the patients with IPF than the control group, and the total annual direct medical cost was higher among patients with IPF ($26 378 vs $14 254).^14,15^

Previous estimates of the economic burden of IPF were based on data collected prior to 2011. Given the introduction of antifibrotic therapies in 2014 and subsequent advancements in the management of IPF, updated healthcare cost estimates are needed to characterize its current economic impact and inform policymaking and reimbursement decisions. This study aimed to quantify the HCRU and direct healthcare costs for patients with IPF in the US with commercial or Medicare Advantage Prescription Drug (MAPD) health plan coverage, compared with a matched population of individuals without IPF. The goal was to assess total healthcare costs and HCRU incurred by patients with IPF and demonstrate their relative magnitude compared with a demographically similar population.

## METHODS

This retrospective observational study used administrative claims and enrollment data from the Optum Research Database (ORD) for the period of 01 January 2016 through 31 December 2023 (study period). Patients with IPF were identified from 01 January 2017 through 01 October 2023 (patient identification period). The study employed variable-length follow-up, which ended at the earliest occurrence of health plan disenrollment, death, or end of the study period.

### Data Source

The ORD is a fully deidentified and Health Insurance Portability and Accountability Act (HIPAA)–compliant claims database that comprises medical and pharmacy claims data (including linked enrollment) from 1993 to present.

### Human Subject Protection

Institutional review board approval or waiver of approval was not required for this study because the study data were secondary and de-identified in accordance with the US Department of Health and Human Services Privacy Rule’s requirements for deidentification codified at 45 CFR § 164.514(b).

### Study Population

This study included a cohort of newly diagnosed patients with IPF and a demographically matched comparator cohort. To be included in the IPF cohort, patients had to have at least 2 claims with an *International Classification of Diseases, Tenth Revision, Clinical Modification* (ICD-10-CM) code for IPF (J84.112) in any claim position during the follow-up period on different dates within 365 days of each other. The index date was defined as the first date with a diagnosis code for IPF during the identification period. All patients in the IPF cohort were adults (aged ≥18 years as of the year of the index date) with continuous enrollment with medical and pharmacy benefits for at least 12 months prior to the index date (baseline period) and for at least 3 months following and including the index date (minimum follow-up period). Patients with any medical claims with a diagnosis code for IPF in any position during the baseline period were excluded. Additionally, patients with any medical claims for other types of ILDs before the second IPF diagnosis during follow-up, or up to 6 months after the second IPF diagnosis during follow-up (or up to the end of the follow-up period, whichever occurred first) were excluded. Other types of ILDs included ILDs associated with autoimmune diseases, sarcoidosis, hypersensitivity pneumonitis, and idiopathic interstitial pneumonia. Lastly, patients with missing or unknown age, sex, geographic region, or insurance type were excluded.

A random sample of demographically matched patients was included as a comparator cohort. Patients were eligible for the comparator cohort if they had no medical claims with a diagnosis code for IPF or fibrosis in any claim position during the study period, were aged at least 18 years old during the study period, had continuous enrollment with medical and pharmacy benefits for at least 15 months during the study period, and had overlapping enrollment with the index month and year of at least 1 patient in the IPF cohort. The comparator cohort was matched in a 4:1 ratio to patients in the IPF cohort based on insurance type, age group, sex, US geographic region, race/ethnicity, and enrollment during the same month and year as the index month and year of a patient with IPF. The index date for patients in the comparator cohort was defined as the index date of the corresponding matched patient with IPF and required at least 12 months of continuous enrollment prior to the index date and at least 3 months of continuous enrollment following and including the index date. The comparator cohort was matched at a ratio of 4:1 to increase statistical power and precision, which was made possible by the ample availability of potential comparators in the ORD. The number of comparators was capped at 4, since there are minimal gains in statistical efficiency in matching at higher ratios.^16^ All individuals meeting the criteria for the comparator cohort were eligible for matching; random selection was used when more than 4 eligible comparators were identified for a given patient with IPF. Demographic matching was employed to ensure some baseline comparability between the cohorts while preserving the natural distribution of comorbidities of general health plan populations for the comparator cohort.

### Study Variables

Demographics captured during the baseline period included the index year, age, sex, insurance type (commercial or MAPD), and region. Race and ethnicity were captured from the linked consumer sociodemographic data and categorized as follows: Asian, Black, Hispanic, White, or unknown/missing. Baseline comorbid conditions were assessed using diagnosis codes on medical claims to calculate the Quan-Charlson Comorbidity Index (CCI) score and to identify the most prevalent comorbid conditions as defined by the Clinical Classifications Software managed by the Agency for Healthcare Research and Quality, and to identify several specific health conditions of interest regardless of prevalence. Past or present nicotine exposure was defined by the presence of tobacco- or nicotine-related Common Procedure Terminology (CPT) codes, Healthcare Common Procedure Coding System (HCPCS) codes, and ICD-10 procedure and diagnosis codes on medical claims, as well as by National Drug Codes (NDC) and HCPCS codes for cessation medications on pharmacy and medical claims.

Data on medications and nonpharmacological treatments used during baseline and follow-up were collected. Medication use by class was assessed using NDC and HCPCS codes on pharmacy and medical claims for the following classes: antifibrotics, biologic immunomodulatory drugs, calcineurin inhibitors, oral corticosteroids, nonbiologic other immunomodulatory drugs, N-acetylcysteine, and sildenafil. Use of nonpharmacological treatments (supplemental oxygen, pulmonary rehabilitation, lung transplantation) was assessed using procedure, diagnosis, and revenue codes on medical claims. Duration of follow-up was reported, defined as the number of days between the index date and the follow-up period end date. The reasons for end of follow-up were collected and categorized as disenrollment, death, or end of study period.

Binary indicators and counts of HCRU were calculated for ambulatory visits (physician office, hospital outpatient), ER visits, inpatient admissions, and pharmacy fills for all drugs for all causes during the baseline period and follow-up period. The number of all-cause inpatient stays and the length of all-cause inpatient stays (days) were reported. All-cause healthcare costs were computed as the sum of health plan and patient paid amounts during the baseline and follow-up periods and presented as weighted per-patient per-month (wPPPM) to account for the variable follow-up. Reported costs reflect the actual amount paid by the health plan and patient to the providers rather than an estimated cost. Total costs were calculated and presented in categories of pharmacy costs and medical costs. Medical costs included subcategories of ambulatory (physician office, hospital outpatient), emergency, inpatient, and other medical. Costs were adjusted for inflation using the annual medical care component of the Consumer Price Index (CPI) to reflect 2023, the most recent year for which the annual CPI was available^17^

Medical HCRU and costs were defined as respiratory-related if the claim had an ICD-10-CM diagnosis code for a respiratory condition (J00.xx-J99.xx) in the primary position or a procedure code for imaging testing (ie, chest radiography, CT scan, or HRCT scan). Time to first hospitalization was calculated as the number of days between the index date and the first subsequent inpatient claim for a hospital stay for all causes and for a respiratory-related condition.

### Analyses

**Descriptive analyses:** All variables were analyzed descriptively, and results were reported by cohort (IPF vs comparator), both overall and stratified by insurance type (commercial and MAPD). Bivariate comparisons of outcomes and other study variables between the 2 cohorts were evaluated using either Student’s t-test or Wilcoxon rank-sum test for continuous variables, and chi-square test for categorical variables. Counts and percentages were provided for categorical variables. Means, standard deviations (SD), medians, and interquartile ranges were calculated for continuous variables. Outcomes (HCRU and costs) were weighted by the duration of observation time to account for varying lengths of follow-up.

**Kaplan-Meier analysis**: Kaplan-Meier analysis was used to evaluate time to first hospitalization; the median time to event and the 95% confidence interval (CI) around the median were reported.

**Multivariable analyses**: All-cause and respiratory-related wPPPM total healthcare costs were modeled using generalized linear models with a gamma distribution and log link to estimate the adjusted cost ratios between cohorts and 95% CIs. Time to all-cause and respiratory-related inpatient visits were modeled using Cox proportional hazards regression to estimate adjusted hazard ratios between cohorts and 95% CIs. Covariates included in the adjusted models were nonrespiratory comorbidities that had a standardized mean difference in baseline prevalence of greater than 10.0% between the IPF and general comparator cohort, with subsequent resolution of conditions with collinear relationships. Additionally, all-cause inpatient stays and use of select medications during baseline were included as covariates to adjust, in part, for underlying differences in serious illness burden.

## RESULTS

### Patient Population

A total of 7670 patients met the study selection criteria for the IPF cohort and 30 680 individuals for the matched comparator cohort. Given the finding that 88 patients in the IPF cohort had claims for lung transplantation during the baseline period and that, clinically, patients post-transplant would no longer be considered as having IPF, these patients were removed from the analysis population, along with the corresponding 352 matched comparators. For the final analysis, 7582 patients were included in the IPF cohort and 30 328 in the matched comparator cohort, which comprised 10.4% commercially insured (IPF cohort, n = 786; comparator cohort, n = 3144) and 89.6% with MAPD coverage (IPF cohort, n = 6796; comparator cohort, n = 27 184) (**Supplemental Figure S1**). Each population comprised exactly 4 comparators per patient with IPF.

### Baseline Characteristics

**Demographics**: The mean (SD) age for the IPF cohort was 76.1 (8.6) years, with patients most commonly in the age group of 70 to 79 years (42.6%) (**Table 1**). The majority was male (58.7%) and patients most often resided in the southern US (48.6%). Most patients were White (71.7%), followed by 12.1% Hispanic, 8.9% Black, 3.2% Asian, and 4.2% unknown/missing race/ethnicity (**Table 1**). For commercially insured patients in the IPF cohort, the mean (SD) age was 66.4 (11.2) years, and the majority was male (67.4%) and White (75.6%) (**Table 1**). For the IPF patients in the MAPD population, the mean (SD) age was 77.2 (7.4) years, and the majority was male (57.7%) and White (71.3%) (**Table 1**). Because the IPF cohort and comparator cohort were exact-matched, there were no differences between the cohorts in the overall population on the demographic characteristics used for matching **(Table 1)**.

**Table 1.** Demographic Characteristics

	**Overall**		**Commercial**		**MAPD**	
**IPF Cohort (n = 7582)**	**Comparator Cohort (n = 30 328)**	**IPF Cohort (n = 786)**	**Comparator Cohort (n = 3144)**	**IPF Cohort (n = 6796)**	**Comparator Cohort (n = 27 184)**	
Age in years, mean (SD)	76.1 (8.6)	76.0 (8.4)	66.4 (11.2)	66.3 (11.1)	77.2 (7.4)	77.1 (7.3)
Age group^a^, years n (%)						
18-49	55 (0.7)	220 (0.7)	41 (5.2)	164 (5.2)	14 (0.2)	56 (0.2)
50-59	231 (3.0)	924 (3.0)	142 (18.1)	568 (18.1)	89 (1.3)	356 (1.3)
60-69	1234 (16.3)	4936 (16.3)	338 (43.0)	1352 (43.0)	896 (13.2)	3584 (13.2)
70-79	3233 (42.6)	12 932 (42.6)	146 (18.6)	584 (18.6)	3087 (45.4)	12 348 (45.4)
≥80	2829 (37.3)	11 316 (37.3)	119 (15.1)	476 (15.1)	2710 (39.9)	10 840 (39.9)
Sex^a^, n (%)						
Female	3133 (41.3)	12 532 (41.3)	256 (32.6)	1024 (32.6)	2877 (42.3)	11 508 (42.3)
Male	4449 (58.7)	17 796 (58.7)	530 (67.4)	2120 (67.4)	3919 (57.7)	15 676 (57.7)
Region^a^, n (%)						
Midwest	1909 (25.2)	7636 (25.2)	202 (25.7)	808 (25.7)	1707 (25.1)	6828 (25.1)
Northeast	1031 (13.6)	4124 (13.6)	67 (8.5)	268 (8.5)	964 (14.2)	3856 (14.2)
South	3683 (48.6)	14 732 (48.6)	360 (45.8)	1440 (45.8)	3323 (48.9)	13 292 (48.9)
West	959 (12.6)	3836 (12.6)	157 (20.0)	628 (20.0)	802 (11.8)	3208 (11.8)
Race/ethnicity^a^, n (%)						
Asian	243 (3.2)	972 (3.2)	31 (3.9)	124 (3.9)	212 (3.1)	848 (3.1)
Black	672 (8.9)	2688 (8.9)	47 (6.0)	188 (6.0)	625 (9.2)	2500 (9.2)
Hispanic	915 (12.1)	3660 (12.1)	90 (11.5)	360 (11.5)	825 (12.1)	3300 (12.1)
White	5436 (71.7)	21 744 (71.7)	594 (75.6)	2376 (75.6)	4842 (71.2)	19 368 (71.2)
Unknown/missing	316 (4.2)	1264 (4.2)	24 (3.1)	96 (3.1)	292 (4.3)	1168 (4.3)
Index year^a^						
2017	1014 (13.4)	4056 (13.4)	142 (18.1)	568 (18.1)	872 (12.8)	3488 (12.8)
2018	1133 (14.9)	4532 (14.9)	143 (18.2)	572 (18.2)	990 (14.6)	3960 (14.6)
2019	1196 (15.8)	4784 (15.8)	163 (20.7)	652 (20.7)	1033 (15.2)	4132 (15.2)
2020	930 (12.3)	3720 (12.3)	103 (13.1)	412 (13.1)	827 (12.2)	3308 (12.2)
2021	1167 (15.4)	4668 (15.4)	105 (13.4)	420 (13.4)	1062 (15.6)	4248 (15.6)
2022	1226 (16.2)	4904 (16.2)	83 (10.6)	332 (10.6)	1143 (16.8)	4572 (16.8)
2023	916 (12.1)	3664 (12.1)	47 (6.0)	188 (6.0)	869 (12.8)	3476 (12.8)

Abbreviations: IPF, idiopathic pulmonary fibrosis; MAPD, Medicare Advantage Prescription Drug; SD, standard deviation.

^a^Cohorts were exact matched 4:1 on insurance type, age group, sex, US geographic region, race/ethnicity, and index month/year, resulting in identical distributions for these variables.

**Clinical characteristics, baseline costs, and end of follow-up period:** Overall, the IPF cohort had higher prevalence of all comorbid conditions and a higher mean (SD) CCI score than the comparator cohort (2.3 [2.0] vs 1.4 [1.8]) (**Table 2**). For example, patients in the IPF cohort had higher prevalence of chronic obstructive pulmonary disease (COPD) (47.3% vs 14.1%), pneumonia (26.1% vs 4.4%), cardiovascular disease (48.7% vs 28.5%), type 2 diabetes (37.0% vs 31.1%), and gastroesophageal reflux disease (38.5% vs 21.3%). Similar patterns were observed in the commercial and MAPD cohorts. Overall, patients in the IPF cohort had numerically higher mean (SD) baseline all-cause PPPM total healthcare costs relative to the comparator cohort ($2322 [$5086] vs $1006 [$2068]) and numerically higher mean baseline all-cause HCRU counts in all service categories (**Table 2**). Additionally, patients with IPF more frequently ended follow-up due to death (36.1% vs 13.2%) (**Table 2**).

**Table 2.** Baseline Clinical Characteristics, Baseline Costs, Baseline Treatments, Follow-up Length, and Reason for End of Follow-up

	**Overall**		**Commercial**		**MAPD**	
**IPF Cohort (n = 7582)**	**Comparator Cohort (n = 30 328)**	**IPF Cohort (n = 786)**	**Comparator Cohort (n = 3144)**	**IPF Cohort (n = 6796)**	**Comparator Cohort (n = 27 184)**	
Baseline CCI score, mean (SD)	2.3 (2.0)	1.4 (1.8)	1.7 (1.9)	0.7 (1.3)	2.3 (2.0)	1.4 (1.8)
Nicotine exposure (past or present), n (%)	3181 (42.0)	6114 (20.2)	320 (40.7)	458 (14.6)	2861 (42.1)	5656 (20.8)
Selected respiratory comorbidities, n (%)						
Acute respiratory failure	1189 (15.7)	704 (2.3)	108 (13.7)	30 (1.0)	1081 (15.9)	674 (2.5)
Asthma	1057 (13.9)	1743 (5.7)	126 (16.0)	138 (4.4)	931 (13.7)	1605 (5.9)
Chronic obstructive pulmonary disease	3587 (47.3)	4276 (14.1)	295 (37.5)	217 (6.9)	3292 (48.4)	4059 (14.9)
Obstructive sleep apnea	1738 (22.9)	3196 (10.5)	192 (24.4)	310 (9.9)	1546 (22.7)	2886 (10.6)
Pneumonia	1981 (26.1)	1327 (4.4)	211 (26.8)	77 (22.4)	1770 (26.0)	1250 (4.6)
Respiratory tract infection	2408 (31.8)	5013 (16.5)	243 (30.9)	543 (17.3)	2165 (31.9)	4470 (16.4)
Lower respiratory tract infection	1406 (18.5)	3821 (12.6)	163 (20.7)	435 (13.8)	1243 (18.3)	3386 (12.5)
Upper respiratory tract infection	1485 (19.6)	1852 (6.1)	132 (16.8)	181 (5.8)	1353 (19.9)	1671 (6.1)
Selected other comorbidities, n (%)						
Acute coronary syndrome	3385 (44.6)	7297 (24.1)	273 (34.7)	443 (14.1)	3112 (45.8)	6854 (25.2)
Angina	441 (5.8)	811 (2.7)	42 (5.3)	52 (1.7)	399 (5.9)	759 (2.8)
Atrial fibrillation	1441 (19.0)	4036 (13.3)	99 (12.6)	209 (6.6)	1342 (19.7)	3827 (14.1)
Cardiovascular disease	3691 (48.7)	8648 (28.5)	297 (37.8)	514 (16.3)	3394 (49.9)	8134 (29.9)
Chronic kidney disease	1922 (25.3)	6092 (20.1)	93 (11.8)	244 (7.8)	1829 (26.9)	5848 (21.5)
Congestive heart failure	1938 (25.6)	3280 (10.8)	127 (16.2)	125 (4.0)	1811 (26.6)	3155 (11.6)
Deep vein thrombosis	234 (3.1)	648 (2.1)	21 (2.7)	47 (1.5)	213 (3.1)	601 (2.2)
Depression	1486 (19.6)	3913 (12.9)	134 (17.0)	222 (7.1)	1352 (19.9)	3691 (13.6)
Fatigue	2375 (31.3)	6110 (20.1)	205 (26.1)	379 (12.1)	2170 (31.9)	5731 (21.1)
Gastroesophageal reflux disease	2919 (38.5)	6452 (21.3)	276 (35.1)	470 (14.9)	2643 (38.9)	5982 (22.0)
Hypertension, arterial	5973 (78.8)	21,865 (72.1)	490 (62.3)	1565 (49.8)	5483 (80.7)	20,300 (74.7)
Hypertension, pulmonary	1184 (15.6)	819 (2.7)	98 (12.5)	37 (1.2)	1086 (16.0)	782 (2.9)
Lung cancer	144 (1.9)	183 (0.6)	7 (0.9)	16 (0.5)	137 (2.0)	167 (0.6)
Myocardial infarction	753 (9.9)	1502 (5.0)	50 (6.4)	82 (2.6)	703 (10.3)	1420 (5.2)
Pulmonary embolism	251 (3.3)	328 (1.1)	24 (3.1)	26 (0.8)	227 (3.3)	302 (1.1)
Stroke	749 (9.9)	2379 (7.8)	57 (7.3)	116 (3.7)	692 (10.2)	2263 (8.3)
Type 2 diabetes	2806 (37.0)	9442 (31.1)	228 (29.0)	578 (18.4)	2578 (37.9)	8864 (32.6)
Baseline medications of interest^a^, ≥1 fill, n (%)	3600 (47.5)	5833 (19.2)	389 (49.5)	542 (17.2)	3211 (47.2)	5291 (19.5)
Baseline all-cause PPPM total (medical and pharmacy) costs, mean (SD), $	2322 (5086)	1006 (2068)	3428 (10 938)	875 (2246)	2194 (3857)	1021 (2046)
Baseline hospitalizations: Any all-cause inpatient stay, n (%)	2217 (29.2)	3770 (12.4)	233 (29.6)	236 (7.5)	1984 (29.2)	3534 (13.0)
Baseline nonpharmacological treatments, n (%)						
Lung transplant	0 (0.0)	< 5 (< 1.0)	0 (0.0)	0 (0.0)	0 (0.0)	< 5 (< 1.0)
Supplemental oxygen	2585 (34.1)	1247 (4.1)	220 (28.0)	69 (2.2)	2365 (34.8)	1178 (4.3)
Pulmonary rehabilitation	246 (3.2)	34 (0.1)	40 (5.1)	< 5 (< 1.0)	206 (3.0)	< 34 (< 1.0)
Baseline all-cause PPPM HCRU counts, mean (SD)						
Ambulatory visit	2.62 (2.21)	1.67 (2.02)	2.09 (1.73)	1.04 (1.27)	2.68 (2.26)	1.74 (2.07)
Emergency room visit	0.10 (0.17)	0.05 (0.17)	0.07 (0.12)	0.03 (0.09)	0.10 (0.18)	0.06 (0.18)
Inpatient stay	0.03 (0.07)	0.01 (0.05)	0.04 (0.07)	0.01 (0.03)	0.03 (0.07)	0.02 (0.05)
Length of stays, days	0.34 (1.00)	0.16 (0.88)	0.32 (1.08)	0.07 (0.49)	0.34 (0.99)	0.17 (0.91)
Other medical visit	0.79 (1.03)	0.42 (0.79)	0.71 (1.46)	0.29 (0.83)	0.80 (0.96)	0.43 (0.79)
Pharmacy fill	3.21 (2.54)	2.40 (2.41)	3.08 (2.83)	1.79 (2.03)	3.23 (2.51)	2.47 (2.44)
Duration of follow-up period (days), mean (SD)	751.4 (564.2)	919.8 (643.5)	706.7 (515.6)	835.3 (610.4)	756.5 (569.3)	929.6 (646.6)
Reason for end of follow-up, n (%)						
Death	2740 (36.1)	3997 (13.2)	130 (16.5)	116 (3.7)	2610 (38.4)	3881 (14.3)
Disenrollment from the health plan	1615 (21.3)	8368 (27.6)	458 (58.3)	1928 (61.3)	1157 (17.0)	6440 (23.7)
End of study period	3227 (42.6)	17,963 (59.2)	198 (25.2)	1100 (35.0)	3029 (44.6)	16,863 (62.0)

Abbreviations: CCI, Quan-Charlson Comorbidity Index, IPF, idiopathic pulmonary fibrosis; MAPD, Medicare Advantage Prescription Drug; SD, standard deviation.

^a^Medications of interest included: antifibrotics, biologic immunomodulatory drugs, calcineurin inhibitors, N-acetylcysteine, non-biologic other immunomodulatory drugs, oral corticosteroids, and sildenafil.

### Follow-up Period

**Medications**: In the overall population, oral corticosteroids were the most commonly used medication class among the classes assessed, received by 59.0% of the patients in the IPF cohort and by 29.8% of the comparator cohort (**Table 3**). A similar pattern was observed in both the commercially insured and MAPD populations. Among the IPF cohort, 29.1%, 40.2%, and 27.8% received antifibrotic medications in the overall, commercially insured, and MAPD populations, respectively (**Table 3**).

**Table 3.** Follow-up Pharmacologic and Nonpharmacologic Treatment Use and All-Cause HCRU Visits

	**Overall**		**Commercial**		**MAPD**	
**IPF Cohort (n = 7582)**	**Comparator Cohort (n = 30 328)**	**IPF Cohort (n = 786)**	**Comparator Cohort (n = 3144)**	**IPF Cohort (n = 6796)**	**Comparator Cohort (n = 27 184)**	
Follow-up treatments of interest, n (%)						
Any medication of interest	5376 (70.9)	9754 (32.2)	591 (75.2)	842 (26.8)	4,785 (70.4)	8912 (32.8)
Antifibrotics	2203 (29.1)	0 (0.0)	316 (40.2)	(0.0)	1887 (27.8)	0 (0.0)
Biologic immunomodulatory drugs	271 (3.6)	892 (2.9)	28 (3.6)	68 (2.2)	243 (3.6)	824 (3.0)
Calcineurin inhibitors	130 (1.7)	59 (0.2)	58 (7.4)	7 (0.2)	72 (1.1)	52 (0.2)
N-acetylcysteine	46 (0.6)	22 (0.1)	8 (1.0)	<5 (<1.0)	38 (0.6)	21 (0.1)
Nonbiologic immunomodulatory drugs	446 (5.9)	881 (2.9)	104 (13.2)	68 (2.2)	342 (5.0)	813 (3.0)
Oral corticosteroids	4476 (59.0)	9038 (29.8)	459 (58.4)	777 (24.7)	4017 (59.1)	8261 (30.4)
Sildenafil	83 (1.1)	26 (0.1)	11 (1.4)	14 (0.5)	72 (1.1)	12 (0.0)
Follow-up nonpharmacological treatments, n (%)						
Lung transplant	204 (2.7)	<5 (<1.0)	80 (10.2)	0 (0.0)	124 (1.8)	<5 (<1.0)
Supplemental oxygen	4833 (63.7)	2970 (9.8)	464 (59.0)	142 (4.5)	4369 (64.3)	2828 (10.4)
Pulmonary rehabilitation	999 (13.2)	70 (0.2)	139 (17.7)	5 (0.2)	860 (12.7)	65 (0.2)
All-cause HCRU visits, wPPPM, mean (SD)						
Ambulatory visit	3.09 (2.39)	1.80 (1.87)	2.75 (2.18)	1.09 (1.10)	3.13 (2.41)	1.88 (1.92)
Emergency room visit	0.14 (0.26)	0.07 (0.21)	0.09 (0.16)	0.03 (0.09)	0.14 (0.27)	0.07 (0.21)
Inpatient stay	0.05 (0.09)	0.02 (0.04)	0.05 (0.09)	0.01 (0.03)	0.05 (0.09)	0.02 (0.05)
Length of stays, days	0.57 (1.35)	0.22 (0.74)	0.64 (1.75)	0.11 (0.65)	0.56 (1.29)	0.23 (0.74)
Other medical visit	1.19 (1.28)	0.48 (0.83)	1.32 (2.02)	0.36 (0.84)	1.18 (1.17)	0.49 (0.83)
Pharmacy fill	3.63 (2.58)	2.46 (2.24)	3.92 (2.90)	1.91 (1.98)	3.60 (2.54)	2.51 (2.25)

Abbreviations: HCRU, healthcare resource utilization; IPF, idiopathic pulmonary fibrosis; MAPD, Medicare Advantage Prescription Drug; SD, standard deviation; wPPPM, weighted per-patient per-month.

Counts <5 and percentages based on those counts have been suppressed.

**HCRU counts**: Overall, the mean all-cause wPPPM counts of HCRU during follow-up were significantly higher in the IPF cohort vs the comparator cohort for all HCRU categories (all *P* < .001) (**Table 3**). Mean (SD) wPPPM counts of ambulatory visits were 3.09 (2.39) vs 1.80 (1.87), ER visits 0.14 (0.26) vs 0.07 (0.21), inpatient stays 0.05 (0.09) vs 0.02 (0.04), length of stays 0.57 (1.35) days vs 0.22 (0.74) days, and pharmacy fills 3.63 (2.58) vs 2.46 (2.24) for the IPF cohort vs comparator cohort, respectively (all *P* < .001) (**Table 3**). Similarly, within the commercial and MAPD populations, wPPPM counts of all-cause HCRU were higher for the IPF cohort than the comparator cohort for all HCRU categories (all *P* < .001) (**Table 3**). Mean respiratory-related wPPPM counts of HCRU during follow-up were significantly higher in the IPF vs the comparator cohort for the overall population and for each insurance type (**Supplemental Table S1**).

For all populations, the difference in HCRU counts wPPPM between baseline and follow-up periods was numerically greater for the IPF cohort than the comparator cohort in all service categories (**Table 2, Table 3**). To further illustrate the change in HCRU before and after IPF diagnosis, the proportion of patients with at least 1 HCRU event by site of service during the 12 months before and after the index date is presented for both cohorts in **Supplemental Figure S2**. The difference between baseline and follow-up in the proportion of patients receiving oxygen supplementation or pulmonary rehabilitation was numerically higher for the IPF cohort than the comparator cohort (**Table 2, Table 3**).

**Time to first all-cause hospitalization:** In the Kaplan-Meier analysis of the overall population, cumulative rates of all-cause hospitalization were significantly higher for the IPF cohort than for the comparator cohort during the follow-up period. The median (95% CI) time to first all-cause hospitalization was 1.63 (1.53, 1.69) years for the IPF cohort and 5.05 (4.92, 5.22) years for the comparator cohort (*P* < .001) (**Supplemental Figure S3A**). Significant differences between the IPF and comparator cohorts were also observed for the commercial population (IPF median [95% CI]: 1.84 [1.55, 2.21] years, comparator: median not reached; *P* < .001) and the MAPD population (IPF median [95% CI]: 1.61 [1.51, 1.68] years, comparator: 4.83 [4.69, 4.96] years; *P* < .001) (**Supplemental Figures S3B and S3C**).

**Time to first respiratory-related hospitalization**: Kaplan-Meier analysis of the overall population found that cumulative rates of respiratory-related hospitalization were significantly higher for the IPF cohort during the follow-up period than for the comparator cohort (IPF median [95% CI]: 1.73 [1.66-1.83] years, comparator: median not reached; *P* < .001) (**Supplemental Figure S4A**). Similar results were observed in the commercial and MAPD populations (**Supplemental Figures S4B and S4C**).

**Figure 1.**
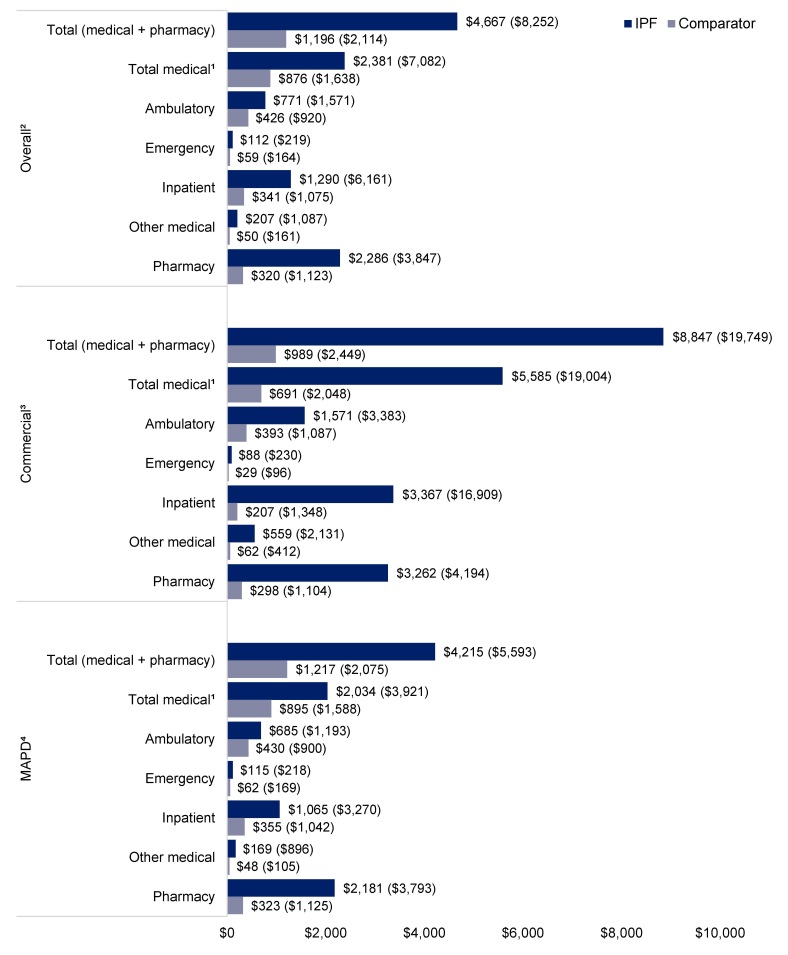
Follow-up wPPPM Mean (SD) All-Cause Healthcare Costs: IPF and Comparator Cohorts Abbreviations: IPF, idiopathic pulmonary fibrosis; MAPD, Medicare Advantage Prescription Drug; SD, standard deviation; wPPPM, weighted per-patient per-month. ^1^Total medical costs comprise ambulatory, emergency, inpatient, and other medical costs. ^2^Overall population: IPF cohort, n = 7582; comparator cohort, n = 30 328. ^3^Commercial population: IPF cohort, n = 786; comparator cohort, n = 3144. ^4^MAPD population: IPF cohort, n = 6796; comparator cohort, n = 27 184.

**Follow-up all-cause healthcare costs**: In the overall population, mean (SD) follow-up all-cause wPPPM total healthcare costs were higher for the IPF cohort than the comparator cohort ($4667 [$8252] vs $1196 [$2114], *P* < .001) (**Figure 1**). The wPPPM mean (SD) all-cause healthcare costs across categories for the IPF cohort compared with the comparator cohort were $2381 ($7082) vs $876 ($1638) for total medical costs, $771 ($1571) vs $426 ($920) for ambulatory costs, $1290 ($6161) vs $341 ($1075) for inpatient costs, and $2286 ($3847) vs $320 ($1123) for pharmacy costs, respectively (all *P* < .001) (**Figure 1**). Inpatient costs accounted for the largest proportion of all-cause total medical costs for the IPF cohort (54% of total medical costs), whereas ambulatory costs accounted for the largest proportion for the comparator cohort (49% of total medical costs) (**Figure 1**).

When stratified by insurance type, mean (SD) all-cause wPPPM total healthcare costs for the IPF cohort vs the comparator cohort, respectively, were $8847 ($19 749) vs $989 ($2449) (*P* < .001) for the commercial population and $4215 ($5593) vs $1217 ($2075) (*P* < .001) for the MAPD population (**Figure 1**). As with the overall population, inpatient costs accounted for the largest proportion of all-cause total medical costs for the IPF cohort for both the commercial population (60%) and the MAPD population (52%) (**Figure 1**). Ambulatory costs accounted for the largest proportion of total medical costs for the commercial comparator cohort (57%) and the MAPD comparator cohort (48%) (**Figure 1**).

**Follow-up respiratory-related healthcare costs**: In the overall population, respiratory-related mean (SD) wPPPM total medical costs during follow-up were higher for the IPF cohort than for the comparator cohort ($1694 [$6563] vs $304 [$1032]) and across all categories (all *P* < .001) (**Supplemental Figure S5A**). A similar pattern was observed in the commercially insured population ($4474 [$17 954] vs $185 [$1282]) and the MAPD population ($1394 [$3465] vs $316 [$1002]) (**Supplemental Figures S5B and S5C**) (all *P* < .001). In the overall population, inpatient costs accounted for the largest proportion of respiratory-related medical costs for both the IPF cohort (74%) and comparator cohort (85%).

**Figure 2.**
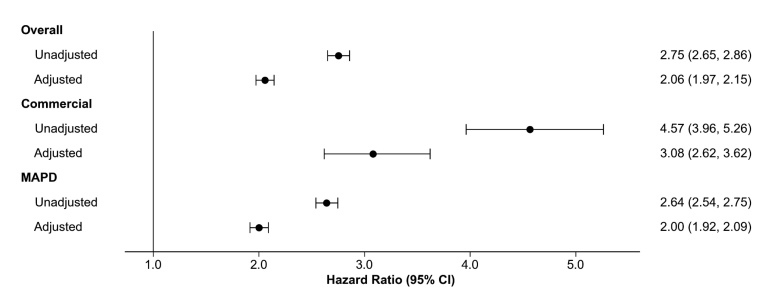
Risk of All-Cause Hospitalization: IPF vs Comparator Cohorts Abbreviations: CI, confidence interval; IPF,  idiopathic pulmonary fibrosis; MAPD,  Medicare Advantage Prescription Drug.

**Risk of all-cause and respiratory-related hospitalization**: In unadjusted Cox proportional hazards regression analysis, the IPF cohort had more than twice the risk of hospitalization for all causes during follow-up than the comparator cohort (hazard ratio, 2.8; 95% CI, 2.65-2.86]) (**Figure 2**). After adjusting for baseline indicators of medication use, prior hospitalization, and nonrespiratory comorbidities, the hazard ratio remained statistically significant; patients in the IPF cohort were 2.1 times (95% CI, 1.97-2.15) more likely to be hospitalized for all causes than the comparator cohort (**Figure 2**; **Supplemental Table S2**). Similar patterns were observed in both the commercial and MAPD cohorts (**Figure 2**).

For respiratory-related hospitalizations, unadjusted analysis demonstrated that the IPF cohort was 3.8 times (95% CI, 3.68-3.98) more likely to be hospitalized for respiratory causes than the comparator cohort (**Supplemental Figure S6**). After covariate adjustment, patients in the IPF cohort remained 2.8 times (95% CI, 2.67-2.92) more likely to be hospitalized for respiratory-related causes than the comparator cohort (**Supplemental Figure S6**; **Supplemental Table S3**). Similar patterns were observed for the commercial and MAPD cohorts.

**Figure 3.**
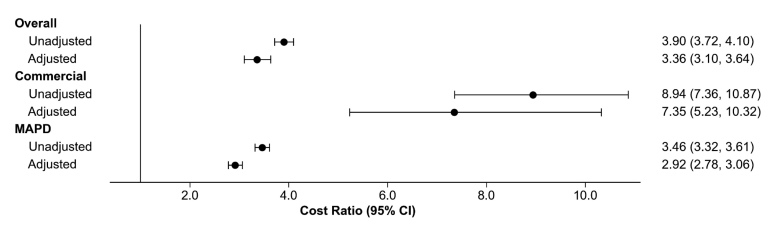
Cost Ratios for All-Cause Total Costs: IPF vs Comparator Cohorts Abbreviations: CI , confidence interval; IPF,  idiopathic pulmonary fibrosis; MAPD,  Medicare Advantage Prescription Drug.

**All-cause and respiratory-related cost ratios**: In unadjusted analysis, all-cause total costs were 3.9 times (95% CI, 3.72-4.10) higher for the IPF cohort than the comparator cohort (**Figure 3**). After adjusting for baseline indicators of medication use, prior hospitalization, and non-respiratory comorbidities, the cost ratio between cohorts remained significant at 3.4 (95% CI, 3.10-3.64) (**Figure 3**; **Supplemental Table S4**). For commercially insured patients, the cost ratio between cohorts was 7.4 (95% CI, 5.23-10.32) for all-cause total costs while the cost ratio for MAPD patients was 2.9 (95% CI, 2.78-3.06) for all-cause total costs (**Figure 3**).

In unadjusted analysis, respiratory-related medical costs were 5.6 times (95% CI, 5.05-6.15) higher for the IPF cohort than the comparator cohort (**Supplemental Figure S7**). After adjusting for baseline indicators of medication use, hospitalization, and non-respiratory comorbidities, the cost ratio between cohorts remained significant at 5.0 times (95% CI, 4.16-6.10) higher for the IPF cohort than the comparator cohort (**Supplemental Figure S7**; **Supplemental Table S5**). Similar patterns were observed for the commercial and MAPD cohorts (**Supplemental Figure S7**).

## DISCUSSION

This retrospective cohort analysis aimed to provide updated estimates of the economic burden among commercially insured or MAPD beneficiaries with IPF in the US. Across analyses, patients with IPF incurred substantially higher HCRU and costs compared with the demographically matched comparator cohort without the disease, with differences primarily driven by hospitalizations. Multivariable analyses that controlled for baseline comorbidities indicated that the risk of all-cause hospitalization during follow-up was approximately twice as high in the IPF cohort and nearly 3 times as high for respiratory-related hospitalization than the comparator cohort. Consistent with these utilization patterns, all-cause wPPPM total healthcare costs for the IPF cohort were more than 3 times higher and respiratory-related total healthcare costs for the IPF cohort were more than 5 times higher than the comparator cohort. Similar patterns were observed when results were stratified by payer type.

The results of this analysis align with prior research demonstrating elevated HCRU and costs for patients with IPF. Wu et al examined healthcare utilization associated with IPF using claims data from 2006-2011.^12^ The findings from Wu et al demonstrated that patients with IPF had higher HCRU including hospitalizations, ED visits, and outpatient visits.^12^ Raimundo et al documented a large healthcare and economic burden associated with IPF and found that, among patients with IPF, the mean per patient number of all-cause outpatient visits in 2011 was 18.5 and the mean per patient number of respiratory-related office visits was 5.7. Annual all-cause costs in 2011 were $59 379 per patient with respiratory-related costs accounting for 36.6% of costs ($21 732).^11^ Two studies by Collard et al reported the HCRU and costs in patients with IPF compared with those without IPF using claims data.^14,15^ For Medicare and commercially insured patients, the Collard studies found that patients with IPF had higher HCRU and costs compared with controls. The Medicare study used data from 2000-2011 while the commercial study used 2001-2008 data. Overall, the present study builds on this body of research by providing contemporary and payer-stratified estimates of the HCRU and cost associated with IPF, thereby further contextualizing the magnitude of the economic burden associated with IPF across insured populations.

While IPF was associated with a higher economic burden, the magnitude of cost differences between cohorts varied by payer type. Several factors contributed to this pattern. First, the MAPD comparator group was older and had a greater comorbidity burden than the commercially insured comparator group, with elevated baseline utilization and costs, which may have narrowed the incremental difference between MAPD IPF and comparator cohorts. Conversely, the younger, healthier composition of the commercial comparator cohort may have amplified the incremental differences with the commercial IPF cohort. Second, commercial insurance plans typically reimburse at higher rates than Medicare Advantage plans, which can increase absolute spending across categories and magnify observed cost differences.^18^ Lastly, benefit design and management practices may differentially influence access to care and treatment, thereby shaping both utilization and costs.

In our analysis, we observed low use of antifibrotic treatments, with modest differences by payer type. This finding aligns with prior studies that have demonstrated limited uptake of the antifibrotic therapies among patients with IPF, despite the therapy being on the market for ten years and guideline recommendation.^19,20^ Notably, high discontinuation rates following treatment initiation have also been documented,^19^ underscoring an area of unmet need and important gap to address to improve IPF care.

### Limitations

This study used a claims-based definition of IPF. The presence of a diagnosis code in claims did not guarantee presence of disease; however, we sought to overcome this potential limitation by requiring a confirmatory diagnosis, thereby limiting misidentification of IPF patients. Patients without an IPF diagnosis in the 12-month baseline were considered newly diagnosed. Patients with IPF may not have sought care for their condition during that 12-month period and may have been misclassified as newly diagnosed; however, this seems unlikely given the frequency with which the IPF cohort received services during follow-up. To be included in this study, patients had to be enrolled in a health plan (ie, commercial or MAPD) during the study period; thus, findings from this study may not be generalizable to all patients with IPF, particularly those without insurance or those without continuous enrollment. This study minimized bias by utilizing exact matching on baseline demographic variables and adjusting for additional covariates, including baseline comorbidities, in regression analyses to examine outcomes of interest; however, residual and unobserved confounding could not be ruled out. There were inherent limitations to the use of a claims database for research, as medical and pharmacy claims were collected for the purpose of payment. Coding errors may have resulted in inaccurate or incomplete data, leading to potential misclassification of variables of interest and bias in research findings. A claim for a prescription was not an indication the medication had been consumed or taken as prescribed. Physician-provided samples, medications taken as part of a clinical trial or over-the-counter medications could not be observed in claims data. Despite these limitations, claims data provided a robust and valuable source for the real-world examination of outcomes. Finally, the observed cost differences between the IPF cohort and the demographically matched comparator cohort should not be interpreted as fully attributable to IPF itself, nor do these results imply that IPF-specific treatment alone would be sufficient to eliminate the observed differences.

## CONCLUSIONS

The study provides contemporary and payer-stratified estimates of economic burden among patients with IPF. Patients with IPF incurred substantially higher HCRU and costs compared with their demographically matched comparators, with differences primarily driven by hospitalizations. The magnitude of burden varied meaningfully by payer type, reflecting differences in baseline risk profiles and reimbursement structures. The hospitalization-driven cost pattern, combined with persistently low antifibrotic uptake, highlights both an unmet clinical need and opportunity to reduce economic burden. While treatment advances may not fully mitigate this elevated burden, our study results suggest potential opportunities for new therapies and management strategies to reduce the burden and offer meaningful improvements for patients, health systems, and payers.

### Competing Interest Disclosure

J.Y. is an employee of BIPI. R.F., A.S., L.L., and B.H.B are employees of Optum, which was paid by BIPI to conduct this study. B.J.B is Professor of Health Services Research at the Mayo Clinic College of Medicine; Endowed Scientific Director, the HSOR Program, and the Section Head, Advanced Analytics, Division of HCDR, the Kern Center.

### Ethical Conduct of Research Statement

Throughout the study, patient privacy was preserved, and researchers complied strictly with all applicable Health Insurance Portability and Accountability Act data management rules and the 1964 Helsinki Declaration and its later amendments or comparable ethical standards.

## Supplementary Material

Online Supplementary Material

## Data Availability

The data contained in our database contains proprietary elements owned by Optum and, therefore, cannot be broadly disclosed or made publicly available at this time. The disclosure of this data to third party clients assumes certain data security and privacy protocols are in place and that the third-party client has executed our standard license agreement which includes restrictive covenants governing the use of the data.
