# Supplementary material for: Real-World Health Care Resource Utilization and Economic Burden Among Patients with Idiopathic Pulmonary Fibrosis in Commercially Insured and Medicare Advantage Populations in the United States
Source: J Health Econ Outcomes Res. 2026 Jun 3;13(1):200–9. doi: 10.36469/001c.161503 (PMC13238604; doi:10.36469/001c.161503)
Supplement: Online Supplementary Material [file jheor_2026_13_1_161503_347492.pdf]

Journal of Health Economics and Outcomes Research

Online Supplementary Material

Real-World Healthcare Resource Utilization and Economic Burden Associated with Idiopathic Pulmonary Fibrosis in Commercially Insured and Medicare Advantage Populations in the United States. *JHEOR*. 2026;13(1):200-209. doi:10.36469/jheor.2026.161503

Figure S1: Patient Sample Selection (IPF Cohort) 1

Table S1: Respiratory-Related HCRU Visits During Follow-up, wPPPM 2

Figure S2: All-Cause HCRU by Service Category During 12-Month Baseline and 12-Month Follow-up Period, Among Individuals with ≥12 Months of Follow-up Continuous Enrollment and ≥1 Matched Comparator Retained per Patient with IPF 3

Figure S3: Kaplan-Meier Analysis of Time to First All-Cause Inpatient Stay, IPF and Comparator Cohorts 7

Figure S4: Kaplan-Meier Analysis of Time to First Respiratory-Related Inpatient Stay, IPF and Comparator Cohorts 10

Figure S5: Figure S5. Follow-up wPPPM Respiratory-Related Healthcare Costs, IPF and Comparator Cohorts 13

Table S2. Proportional Hazards Model of All-Cause Hospitalization with Clustering, Adjusted: Overall Population 16

Figure S6. Risk of Respiratory-Related Hospitalization, IPF vs Comparator Cohorts 17

Table S3. Proportional Hazards Model of Respiratory-Related Hospitalization with Clustering, Adjusted–Overall Population 18

Table S4. Generalized Linear Model With Gamma Distribution of All-Cause wPPPM Total Healthcare Costs, Adjusted – Overall Population 19

Figure S7. Respiratory-Related Costs 20

Table S5. Generalized Linear Model With Gamma Distribution of Respiratory-Related wPPPM Total Medical Costs, Adjusted – Overall Population 21

This supplementary material has been provided by the authors to give readers additional information about their work.

This is an open-access article distributed under the terms of the Creative Commons Attribution 4.0 International License (CCBY-4.0). View this license's legal deed at http://creativecommons.org/licenses/by/4.0 and legal code at http://creativecommons.org/licenses/by/4.0/legalcode for more information.

Figure S1. Patient Sample Selection (IPF Cohort)

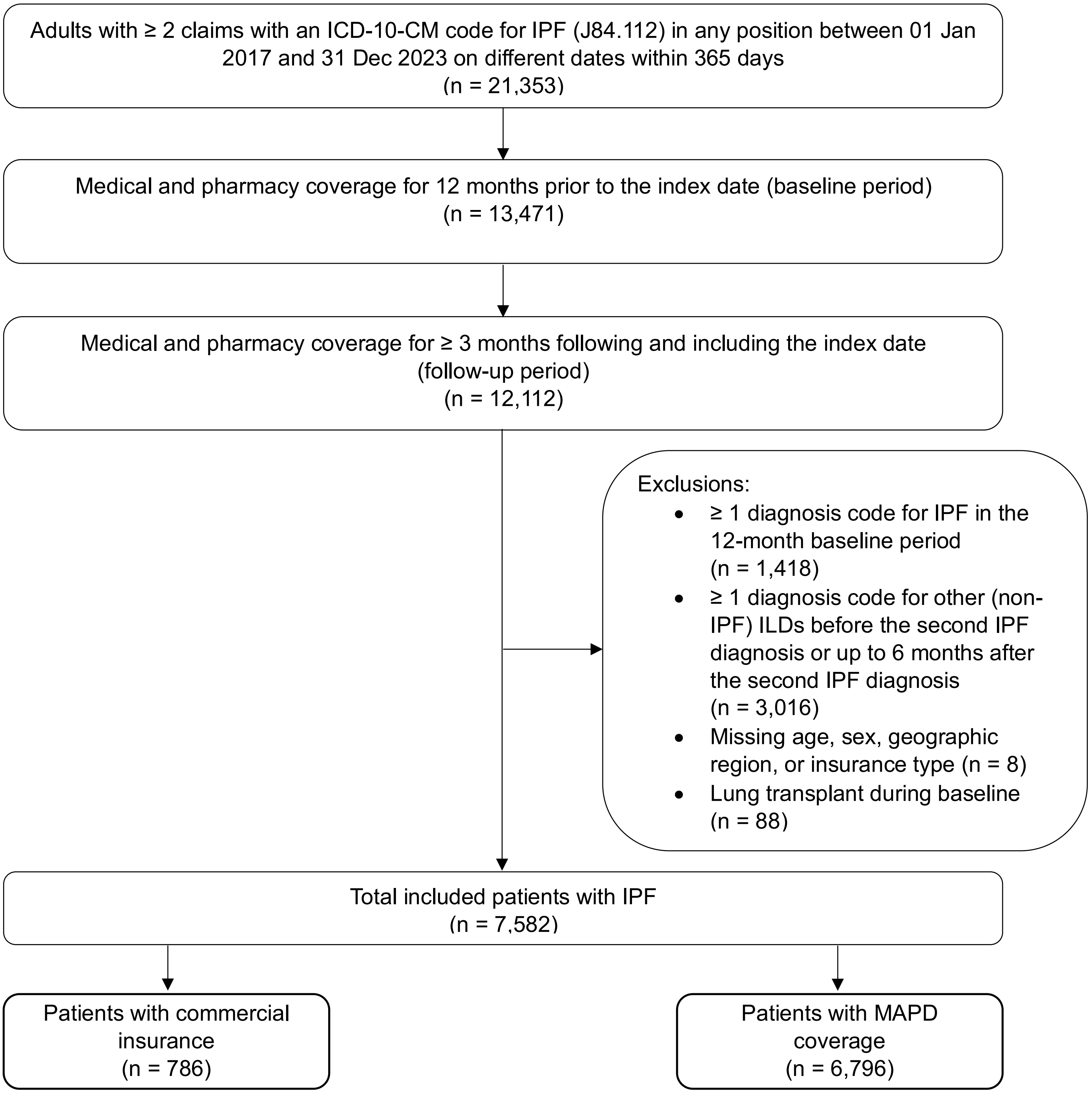

Abbreviations: ICD-10 CM, International Classification of Diseases, Tenth Revision, Clinical Modification; ILD, interstitial lung disease; IPF, idiopathic pulmonary fibrosis; MAPD, Medicare Advantage Prescription Drug.

Table S1. Respiratory-Related HCRU Visits During Follow-up, wPPPM

	Overall		Commercial		MAPD	
	IPF Cohort (n = 7582)	Comparator Cohort (n = 30,328)	IPF Cohort (n = 786)		IPF Cohort (n = 7582)	Comparator Cohort (n = 30,328)
**Respiratory-related HCRU visit counts, wPPPM, mean (SD)**						
Ambulatory visit	1.22 (1.22)	0.16 (0.45)	1.06 (0.97)	0.08 (0.22)	1.23 (1.25)	0.16 (0.47)
Emergency room visit	0.09 (0.16)	0.03 (0.09)	0.06 (0.10)	0.01 (0.03)	0.09 (0.17)	0.03 (0.09)
Inpatient stay	0.05 (0.09)	0.01 (0.03)	0.05 (0.08)	0.01 (0.02)	0.05 (0.09)	0.01 (0.04)
Length of stay, days	0.55 (1.33)	0.16 (0.64)	0.61 (1.75)	0.07 (0.42)	0.55 (1.28)	0.17 (0.65)
Other medical visit	0.58 (0.73)	0.05 (0.28)	0.65 (1.14)	0.04 (0.18)	0.58 (0.67)	0.05 (0.29)

Abbreviations: HCRU, healthcare resource utilization; IPF, idiopathic pulmonary fibrosis; MAPD, Medicare Advantage Prescription Drug; SD, standard deviation; wPPPM, weighted per-patient per-month.

HCRU was defined as respiratory-related if the claim had a diagnosis code for a respiratory condition (J00.xx-J99.xx) in the primary position or a procedure code for imaging testing (ie, chest radiography, high-resolution CT [HRCT] chest).

Figure S2. All-Cause HCRU by Service Category During 12-Month Baseline and 12-Month Follow-up Period, Among Individuals with ≥12 Months of Follow-up Continuous Enrollment and ≥1 Matched Comparator Retained per Patient with IPF

A. Ambulatory Visits, by Population

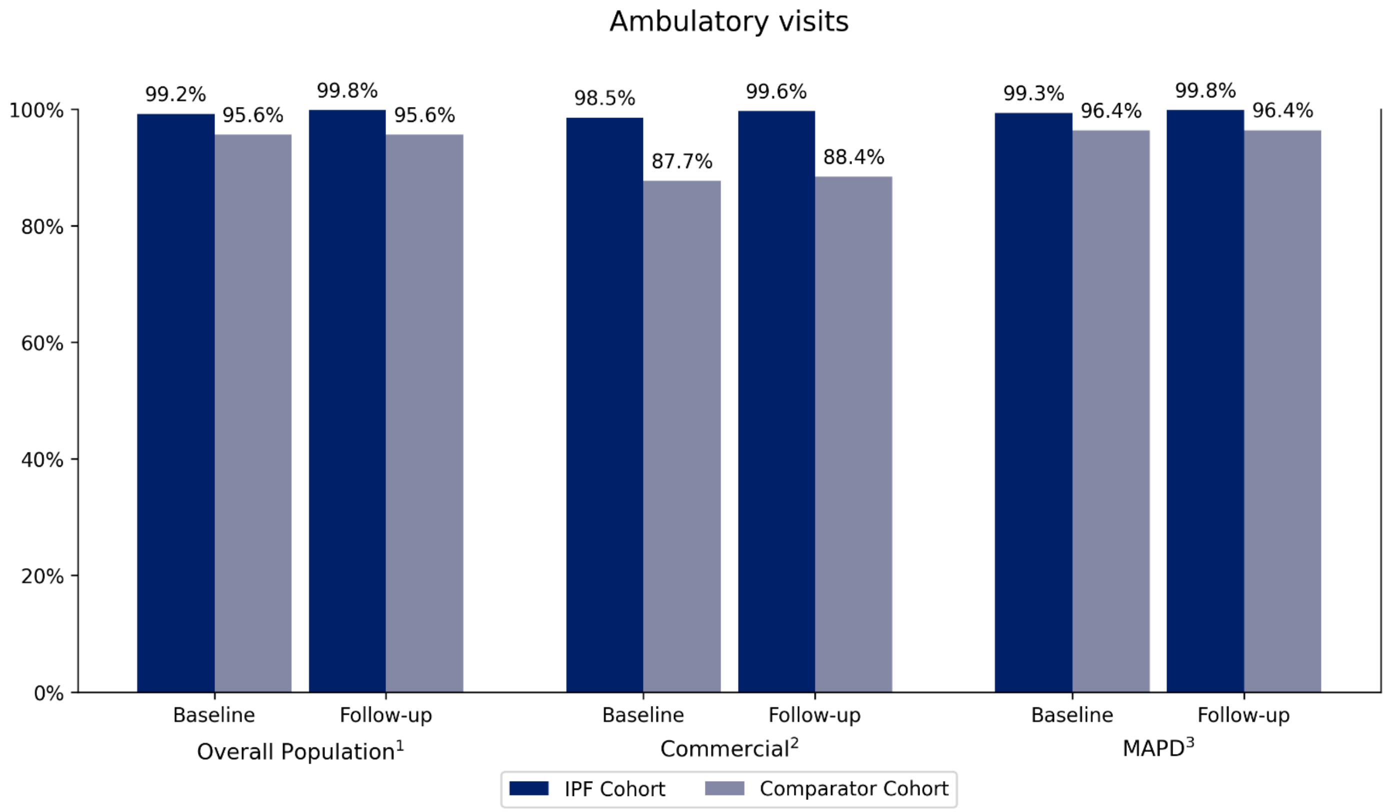

Abbreviations: IPF, idiopathic pulmonary fibrosis; MAPD, Medicare Advantage Prescription Drug.

[1] Overall population: IPF cohort, N = 5199; Comparator cohort, N = 18,131.

[2] Commercial population: IPF cohort, N = 527; Comparator cohort, N = 1649.

[3] MAPD population: IPF cohort, N = 4672; Comparator cohort, N = 16,482.

B. ER Visits, by Population

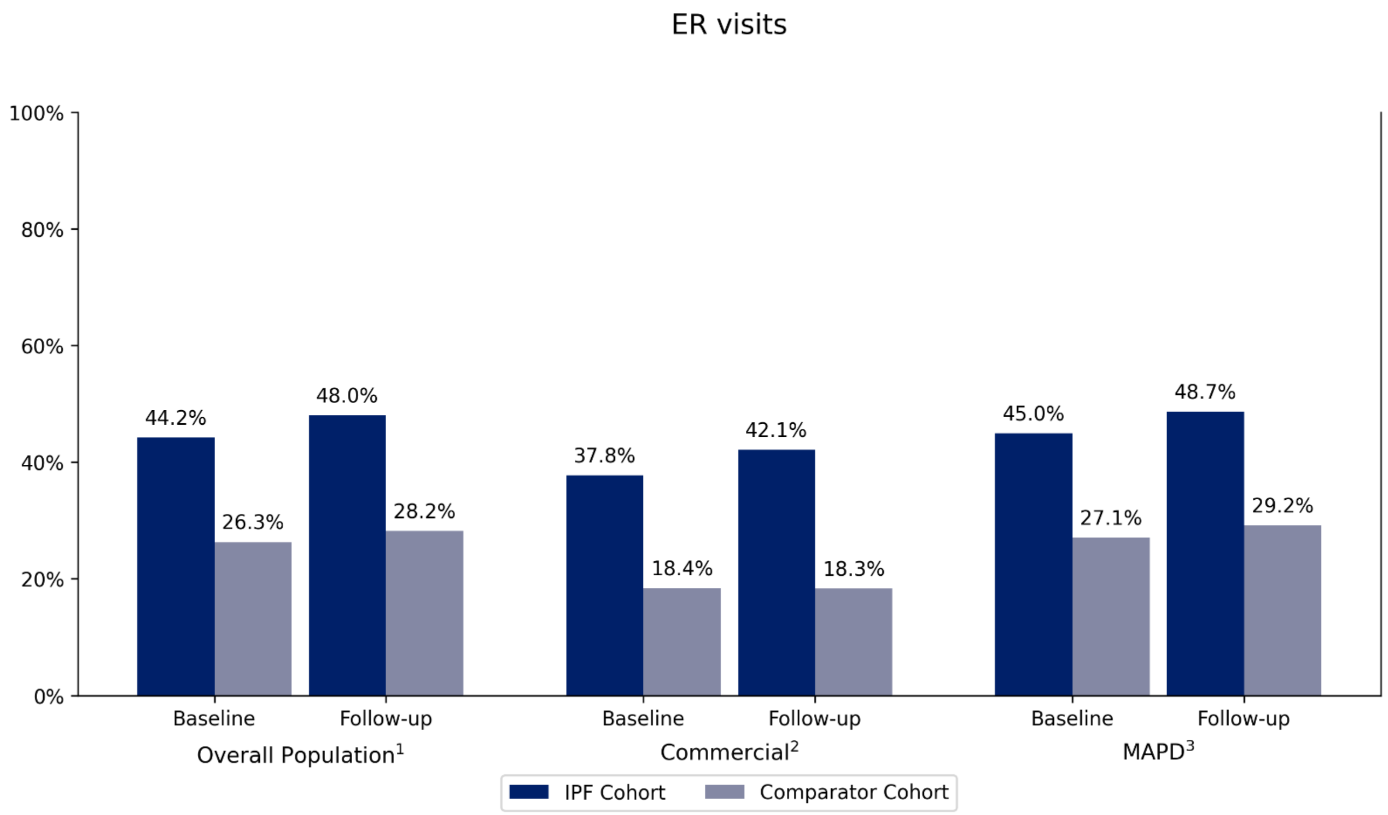

Abbreviations: IPF, idiopathic pulmonary fibrosis; MAPD, Medicare Advantage Prescription Drug.
[1] Overall population: IPF cohort, N = 5199; Comparator cohort, N = 18,131.
[2] Commercial population: IPF cohort, N = 527; Comparator cohort, N = 1649.
[3] MAPD population: IPF cohort, N = 4672; Comparator cohort, N = 16,482.

C. Inpatient Stays, by Population

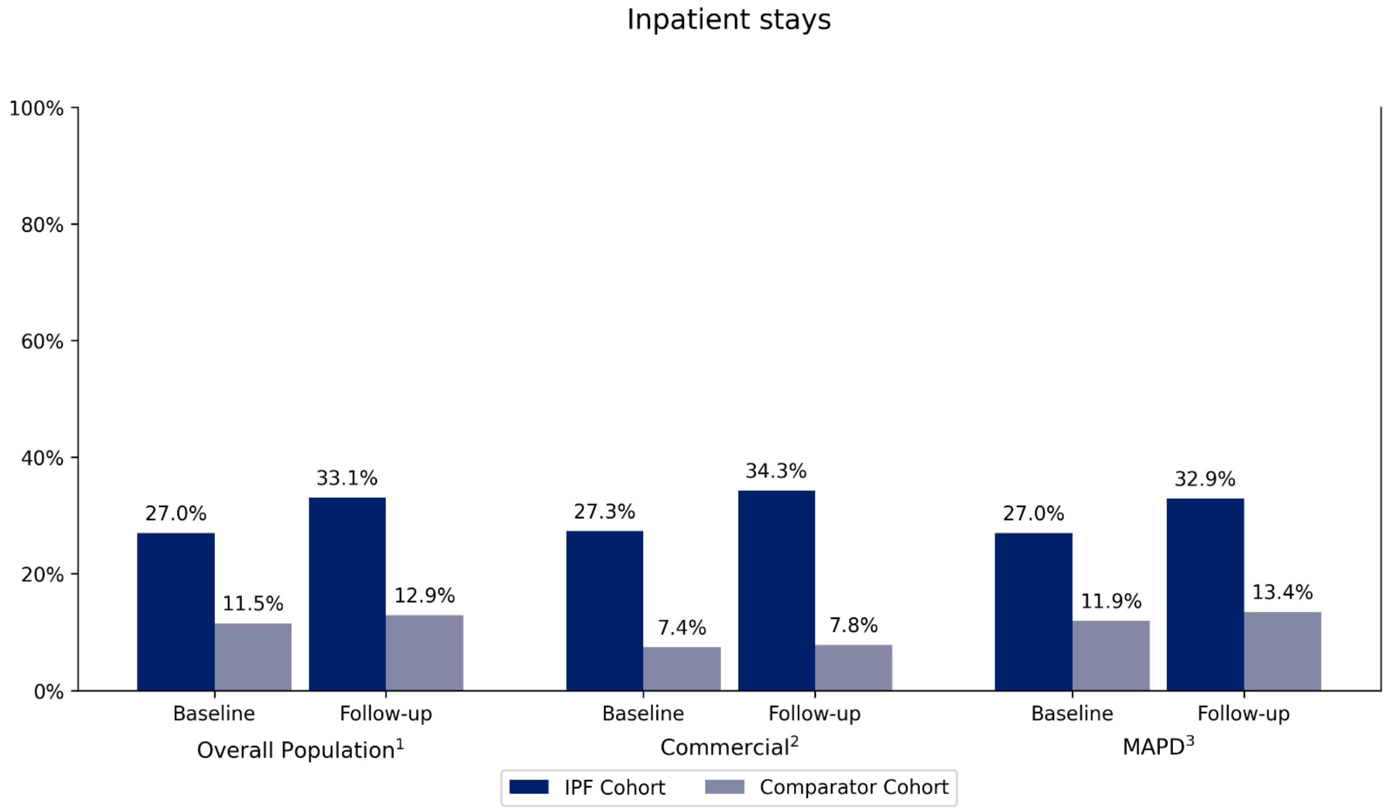

Abbreviations: IPF, idiopathic pulmonary fibrosis; MAPD, Medicare Advantage Prescription Drug.
[1] Overall population: IPF cohort, N = 5199; Comparator cohort, N = 18,131.
[2] Commercial population: IPF cohort, N = 527; Comparator cohort, N = 1649.
[3] MAPD population: IPF cohort, N = 4672; Comparator cohort, N = 16,482.

D. Other Medical Visits, by Population

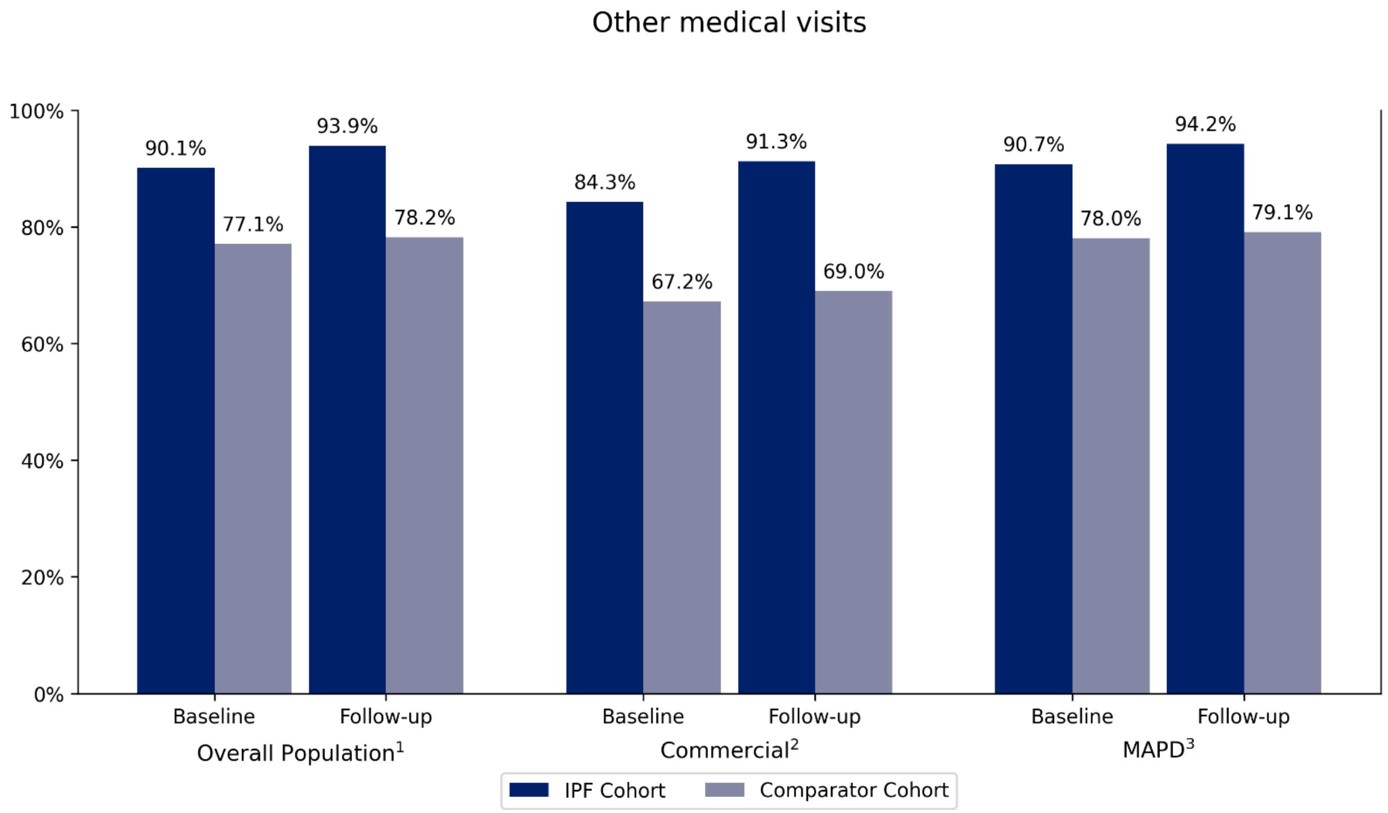

Abbreviations: IPF, idiopathic pulmonary fibrosis; MAPD, Medicare Advantage Prescription Drug.
[1] Overall population: IPF cohort, N = 5199; Comparator cohort, N = 18,131.
[2] Commercial population: IPF cohort, N = 527; Comparator cohort, N = 1649.
[3] MAPD population: IPF cohort, N = 4672; Comparator cohort, N = 16,482.

Figure S3. Kaplan-Meier Analysis of Time to First All-Cause Inpatient Stay, IPF and Comparator Cohorts

A. Overall Population

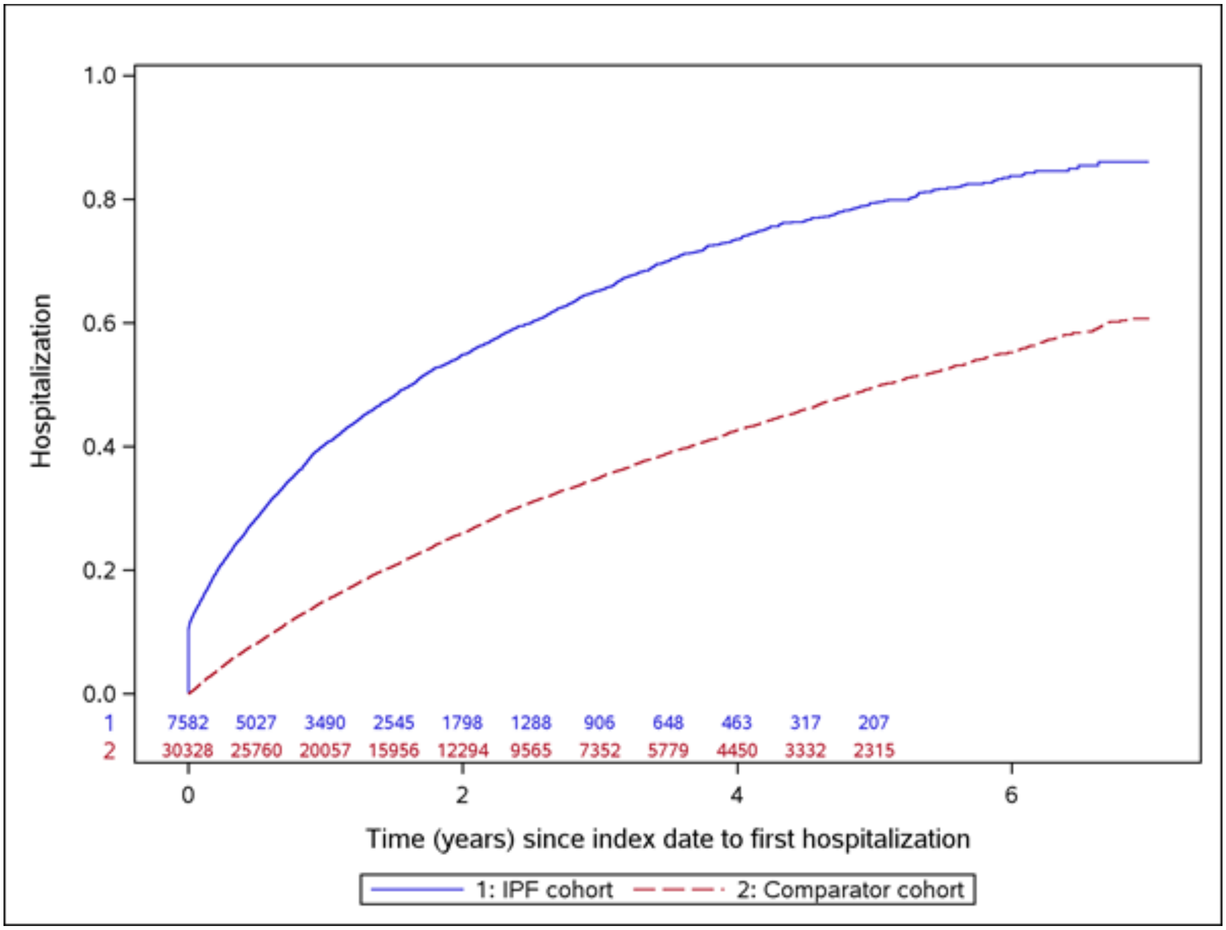

Abbreviation: IPF, idiopathic pulmonary fibrosis.

B. Commercial Population

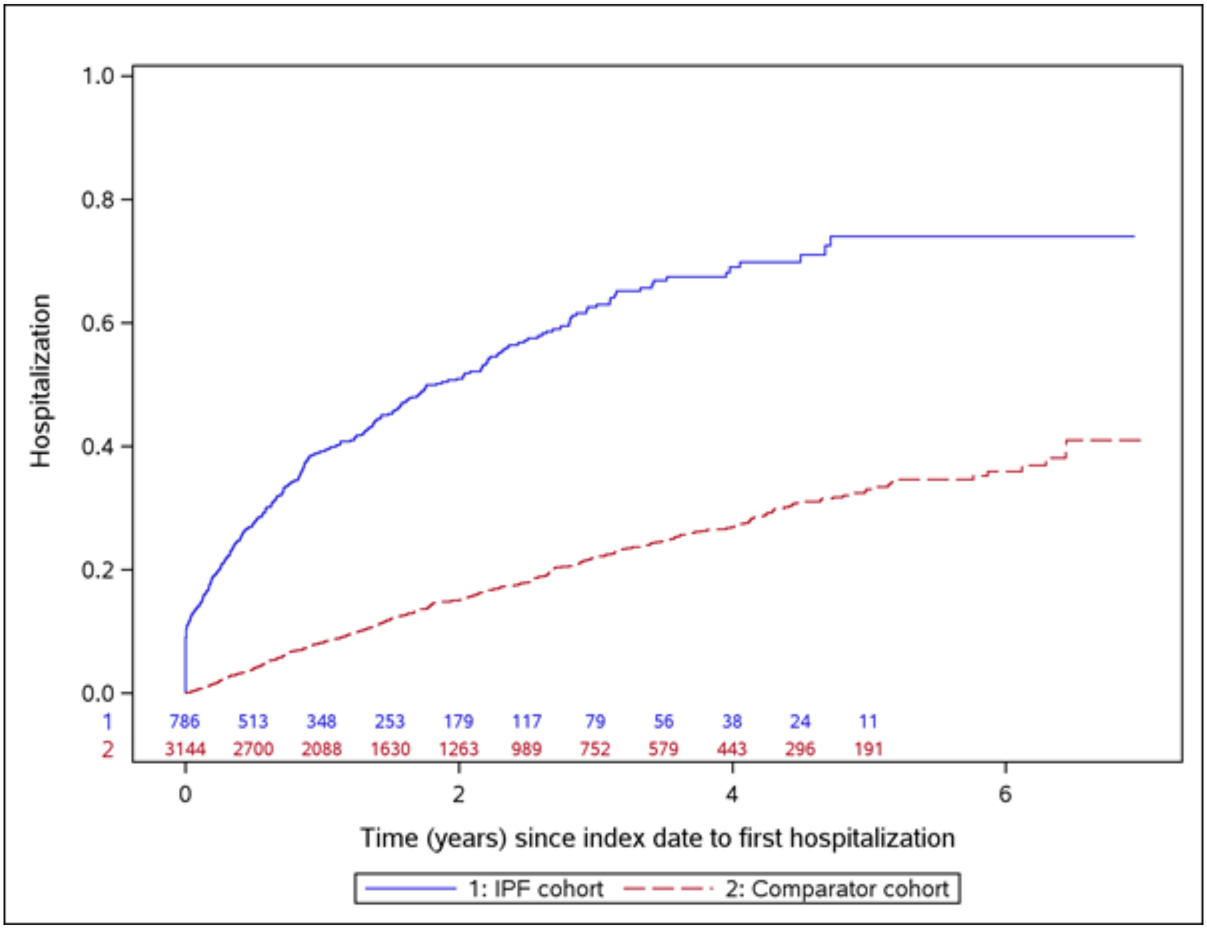

Abbreviation: IPF, idiopathic pulmonary fibrosis.

C. MAPD Population

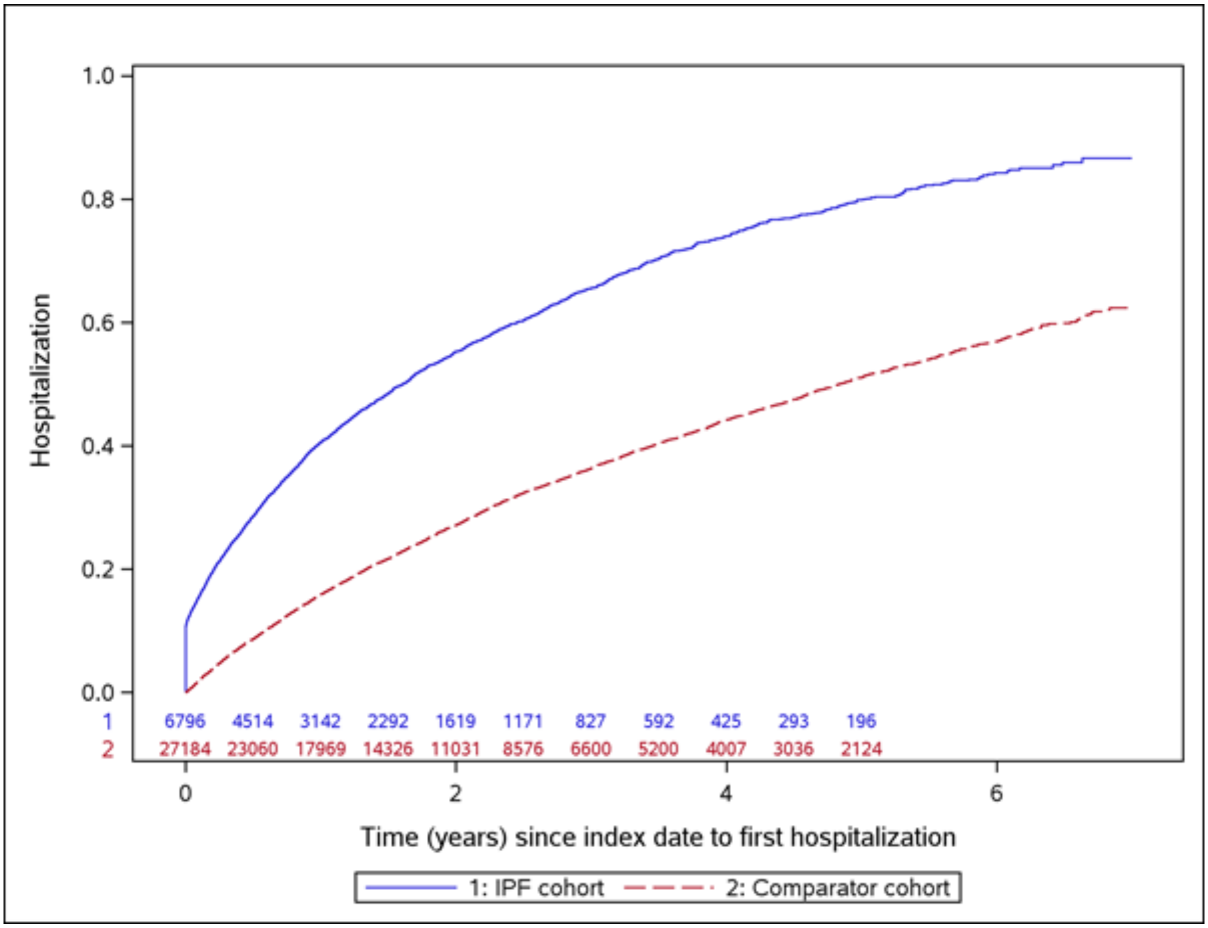

Abbreviations: IPF, idiopathic pulmonary fibrosis; MAPD, Medicare Advantage Prescription Drug.

Figure S4. Kaplan-Meier Analysis of Time to First Respiratory-Related Inpatient Stay, IPF and Comparator Cohorts

A. Overall Population

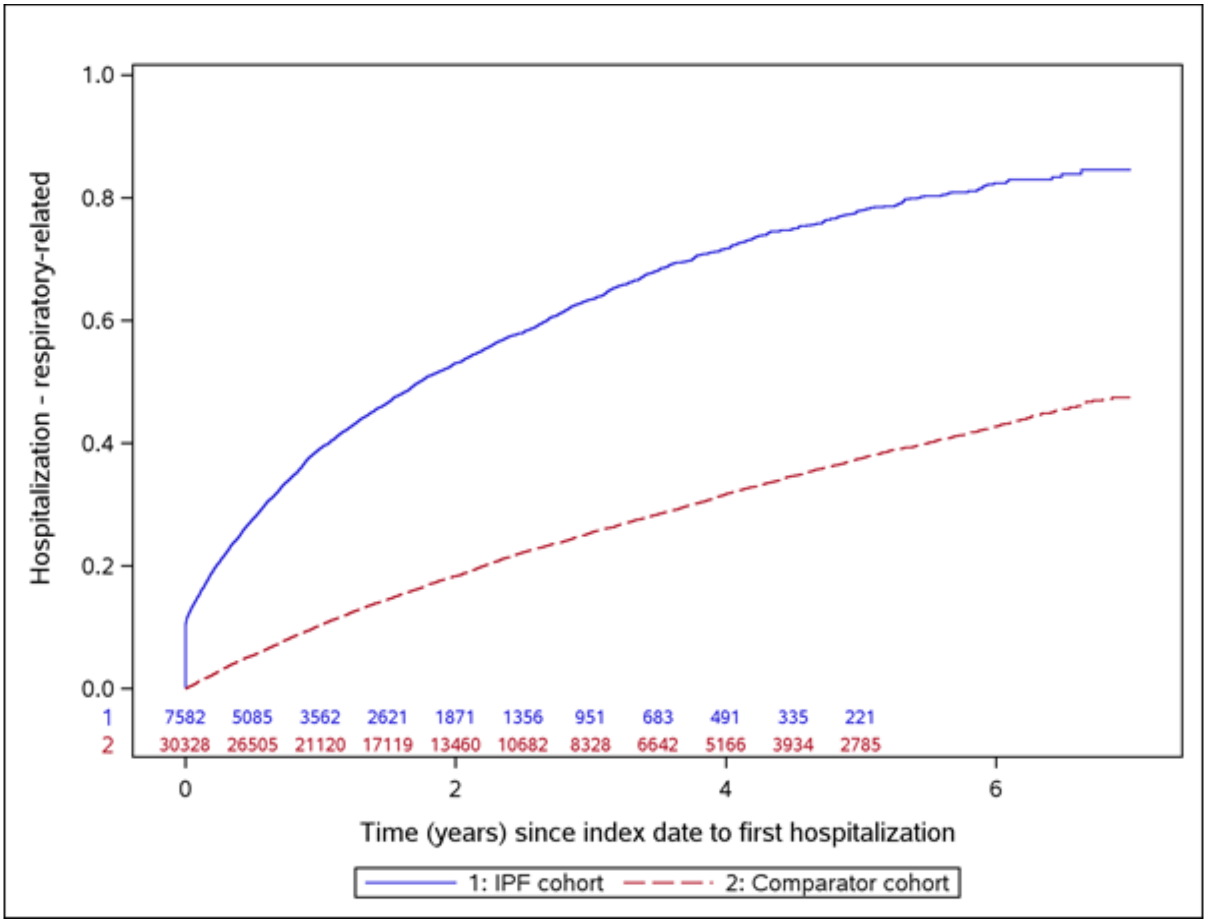

Abbreviation: IPF, idiopathic pulmonary fibrosis.

Hospitalization was defined as respiratory-related if the claim had a diagnosis code for a respiratory condition (J00.xx-J99.xx) in the primary position or a procedure code for imaging testing (ie, chest radiography, high-resolution CT [HRCT] chest).

B. Commercial Population

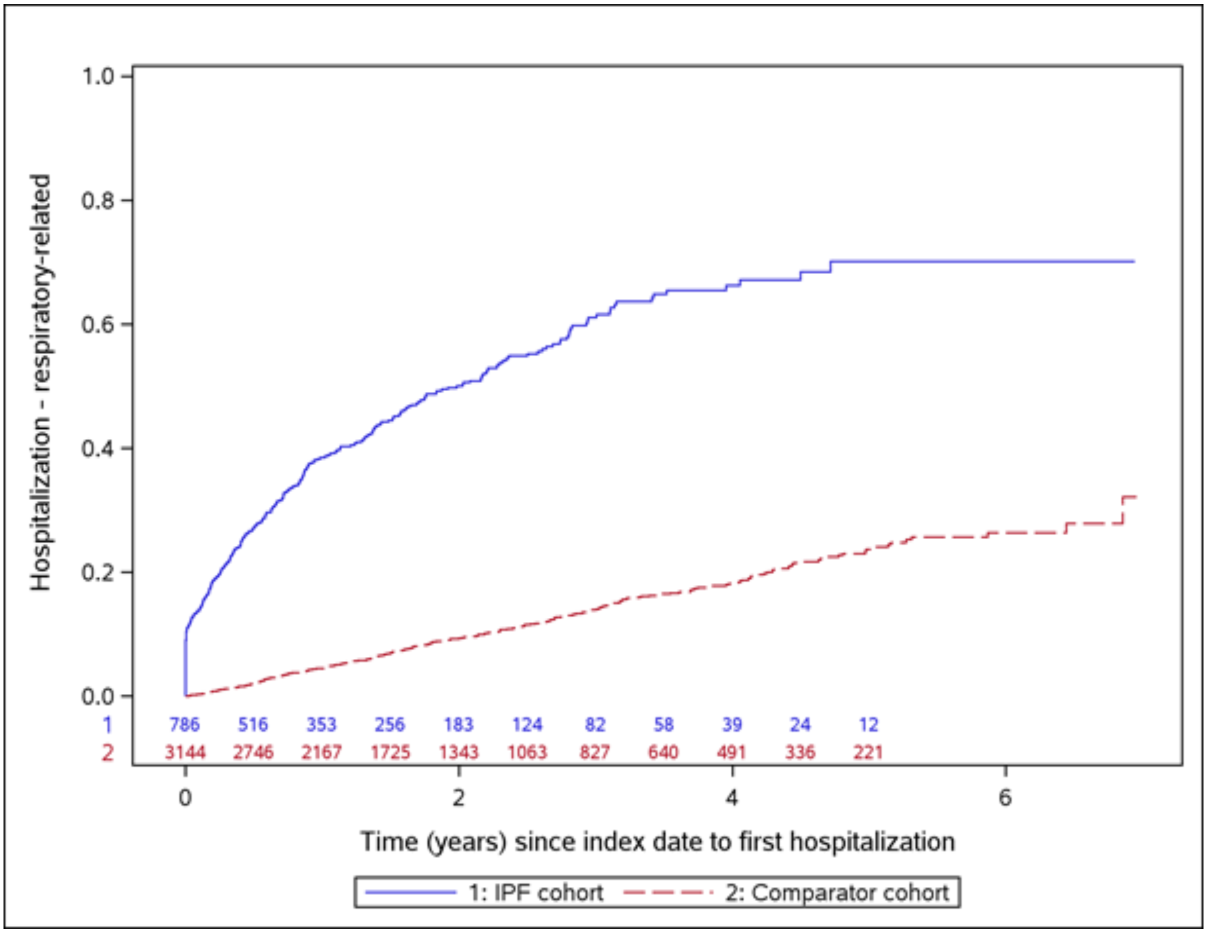

Abbreviation: IPF, idiopathic pulmonary fibrosis.

Hospitalization was defined as respiratory-related if the claim had a diagnosis code for a respiratory condition (J00.xx-J99.xx) in the primary position or a procedure code for imaging testing (ie, chest radiography, high-resolution CT [HRCT] chest).

C. MAPD Population

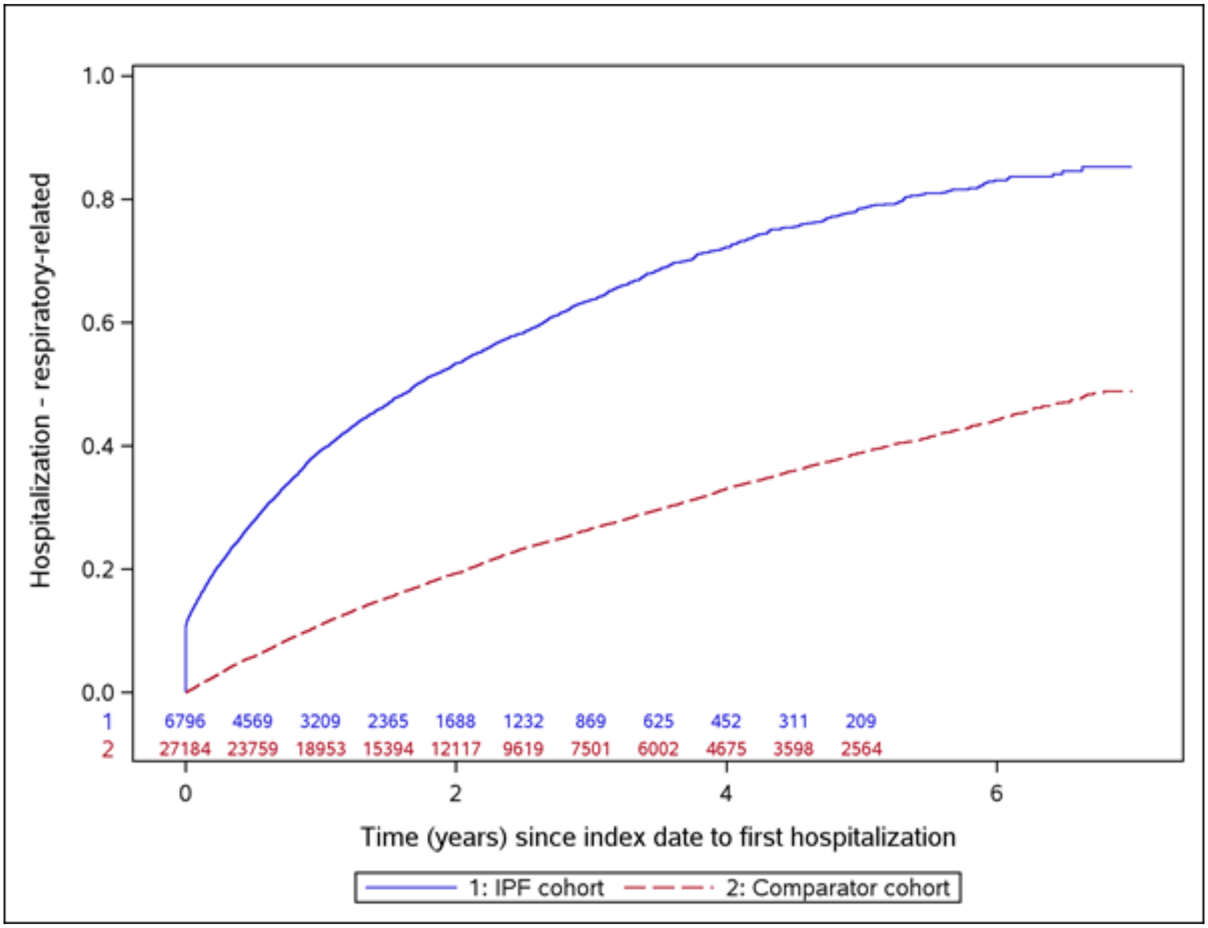

Abbreviation: IPF, idiopathic pulmonary fibrosis.

Hospitalization was defined as respiratory-related if the claim had a diagnosis code for a respiratory condition (J00.xx-J99.xx) in the primary position or a procedure code for imaging testing (ie, chest radiography, high-resolution CT [HRCT] chest).

Figure S5. Follow-up wPPPM Respiratory-Related Healthcare Costs, IPF and Comparator Cohorts[1]

A. Overall Population

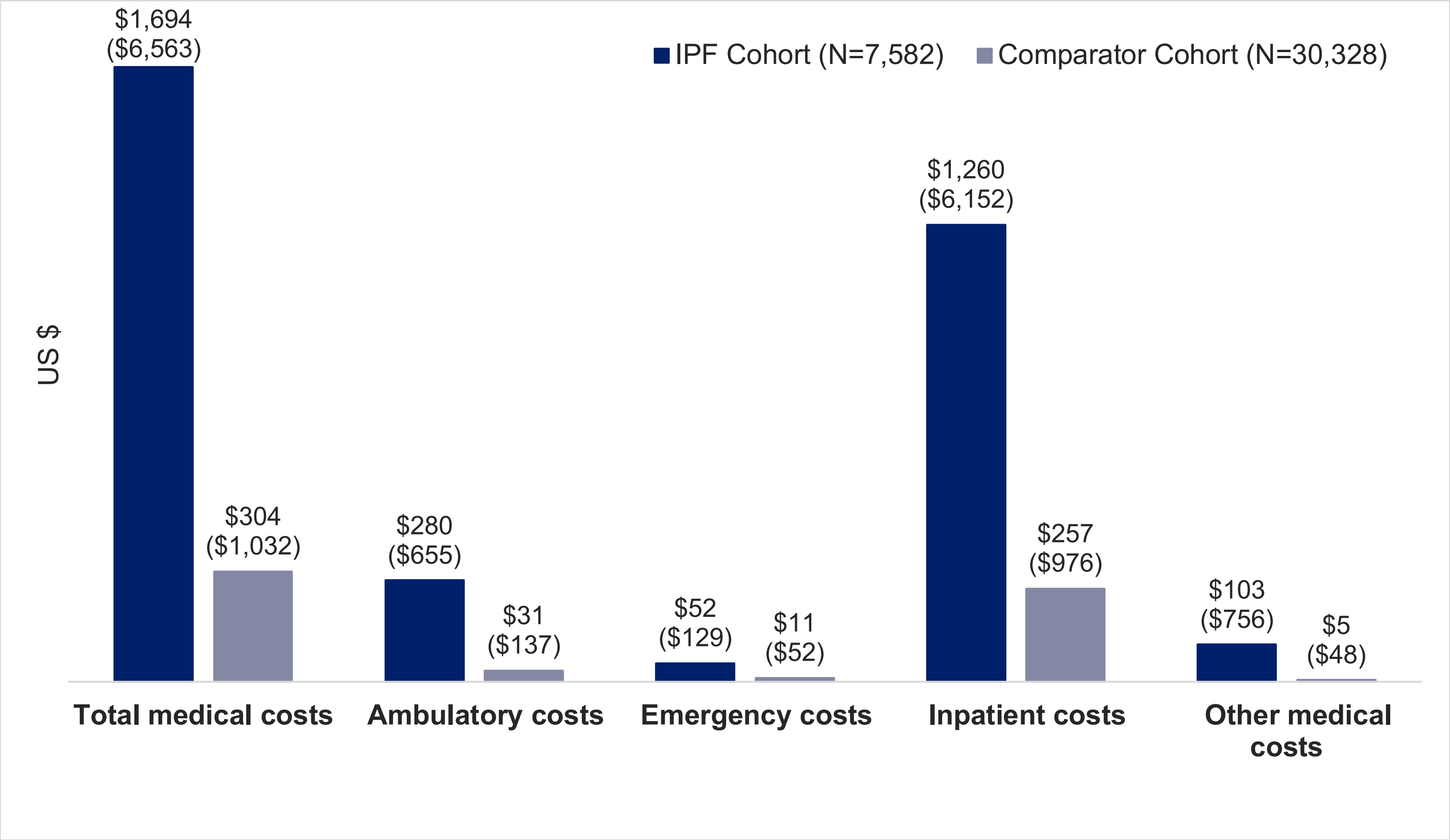

Abbreviations: IPF, idiopathic pulmonary fibrosis; wPPPM, weighted per-patient per-month.

Medical costs were defined as respiratory-related if the claim had a diagnosis code for a respiratory condition (J00.xx-J99.xx) in the primary position or a procedure code for imaging testing (i.e., chest radiography, high-resolution CT [HRCT] chest).

[1]Total medical costs are comprised of ambulatory, emergency, inpatient, and other medical costs.

B. Commercial Population[1]

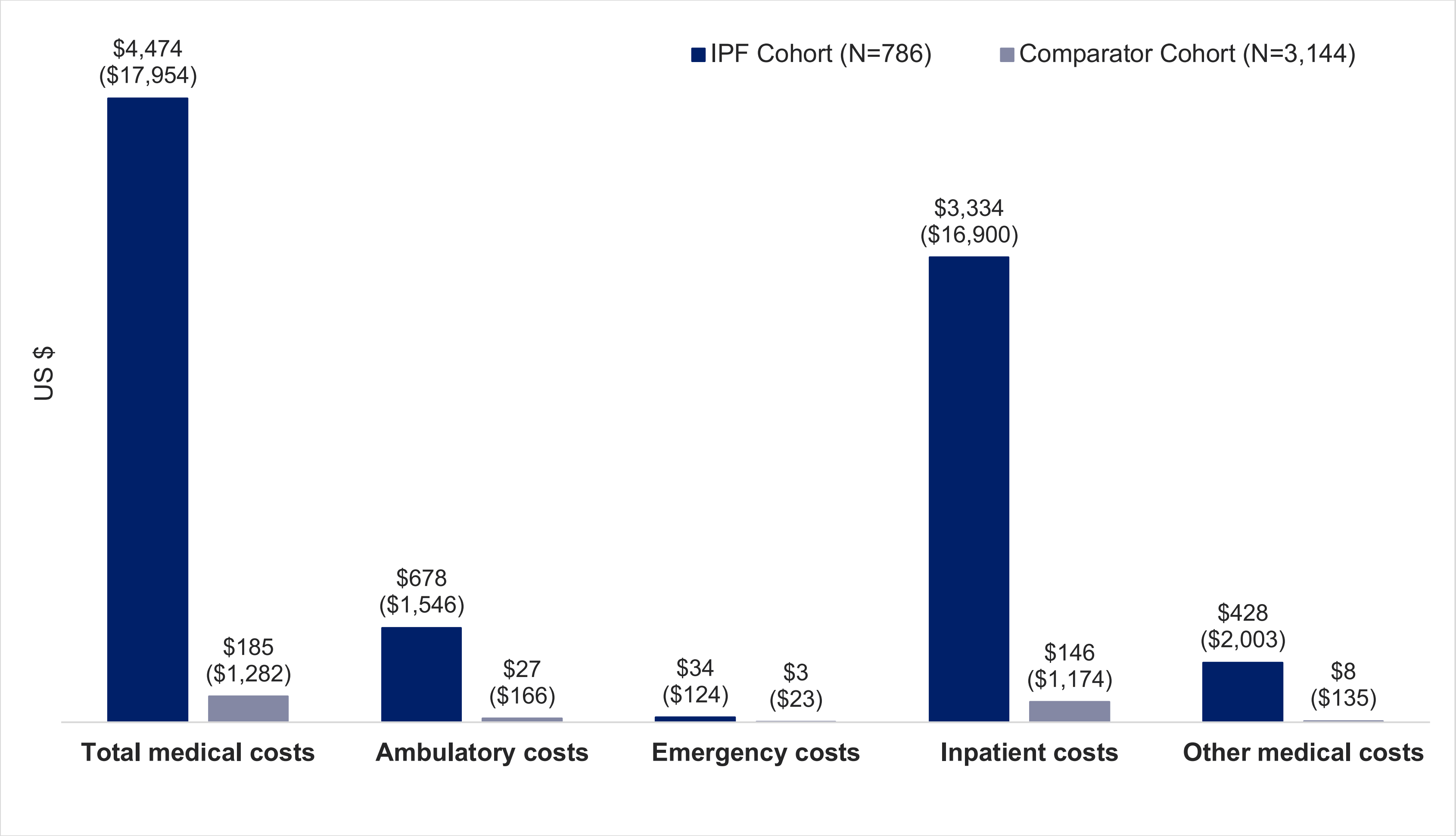

Abbreviations: IPF, idiopathic pulmonary fibrosis; wPPPM, weighted per-patient per-month.

Medical costs were defined as respiratory-related if the claim had a diagnosis code for a respiratory condition (J00.xx-J99.xx) in the primary position or a procedure code for imaging testing (i.e., chest radiography, high-resolution CT [HRCT] chest).

[1]Total medical costs are comprised of ambulatory, emergency, inpatient, and other medical costs.

C. MAPD Population[1]

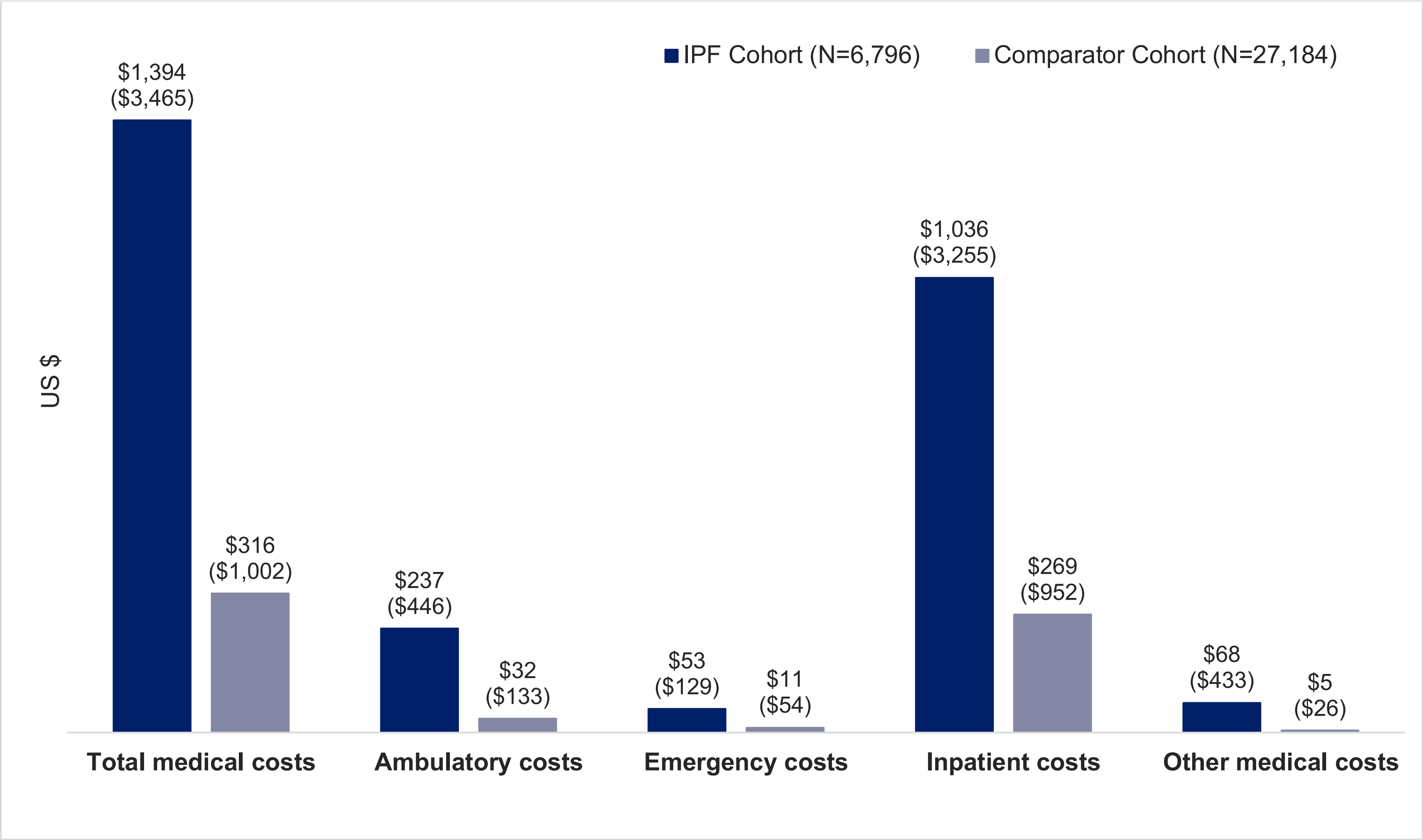

Abbreviations: IPF, idiopathic pulmonary fibrosis; MAPD, Medicare Advantage Prescription Drug; wPPPM, weighted per-patient per-month.

Medical costs were defined as respiratory-related if the claim had a diagnosis code for a respiratory condition (J00.xx-J99.xx) in the primary position or a procedure code for imaging testing (i.e., chest radiography, high-resolution CT [HRCT] chest).

[1]Total medical costs are comprised of ambulatory, emergency, inpatient, and other medical costs.

Table S2. Proportional Hazards Model of All-Cause Hospitalization with Clustering, Adjusted: Overall Population

Independent Variables	All-Cause Hospitalization			
	**Hazard Ratio**	**Lower 95% CI**	**Upper 95% CI**	***P***
Cohort				
Comparator	ref.	–	–	–
IPF	2.06	1.97	2.15	<0.001
Hospitalized during baseline	1.31	1.24	1.38	<0.001
Any medications filled during baseline	1.11	1.07	1.16	<0.001
**Other select comorbidities**				
Acute coronary syndrome (ACS)	1.14	1.09	1.20	<0.001
Angina	0.93	0.85	1.01	0.086
Atrial fibrillation	1.31	1.25	1.37	<0.001
Chronic kidney disease (CKD)	1.16	1.11	1.22	<0.001
Congestive heart failure (CHF)	1.36	1.29	1.43	<0.001
Depression	1.13	1.08	1.19	<0.001
Fatigue	1.12	1.07	1.17	<0.001
Gastroesophageal reflux disease (GERD)	1.04	0.99	1.08	0.104
Hypertension, arterial	1.23	1.17	1.29	<0.001
Lung cancer	1.27	1.07	1.50	0.006
Myocardial infarction	1.09	1.02	1.17	0.013
Pulmonary embolism	1.03	0.90	1.18	0.672
Type 2 diabetes	1.21	1.17	1.26	<0.001
**AHRQ CCS comorbidities**				
Disorders of lipid metabolism	0.81	0.78	0.85	<0.001
Diseases of the heart	1.21	1.16	1.27	<0.001
Immunizations and screening for infectious disease	0.99	0.96	1.03	0.670
Diseases of the urinary system	1.15	1.10	1.20	<0.001
Other nutritional; endocrine; and metabolic disorders	0.97	0.93	1.01	0.090
Other connective tissue disease	1.11	1.07	1.16	<0.001
Diseases of arteries; arterioles; and capillaries	1.14	1.10	1.19	<0.001
Other nervous system disorders	1.16	1.12	1.21	<0.001
Spondylosis; intervertebral disc disorders; other back problems	1.02	0.98	1.06	0.382
Ear conditions	1.00	0.96	1.04	0.959

Observations read = 37,910, Observations used= 37,910.
Overall proportionality test <0.001.
Variable proportionality tests cohort_ana_r1 <0.001, B_IP 0.027, B_ACS 0.003, B_ANGINA 0.593, B_ATRFIB 0.013, B_CKD 0.056, B_CHF 0.997, B_DEPR 0.320, B_FATIGUE 0.478, B_GERD 0.573, B_HTSN 0.408, B_LUNGC 0.200, B_ACSMI 0.745, B_VTEPULEM 0.030, B_DIAB2 0.048, B_AHRQ_27 0.886, B_AHRQ_62 0.021, B_AHRQ_5 0.001, B_AHRQ_87 0.018, B_AHRQ_32 0.894, B_AHRQ_108 0.741, B_AHRQ_64 0.255.

Figure S6. Risk of Respiratory-Related Hospitalization, IPF vs Comparator Cohorts

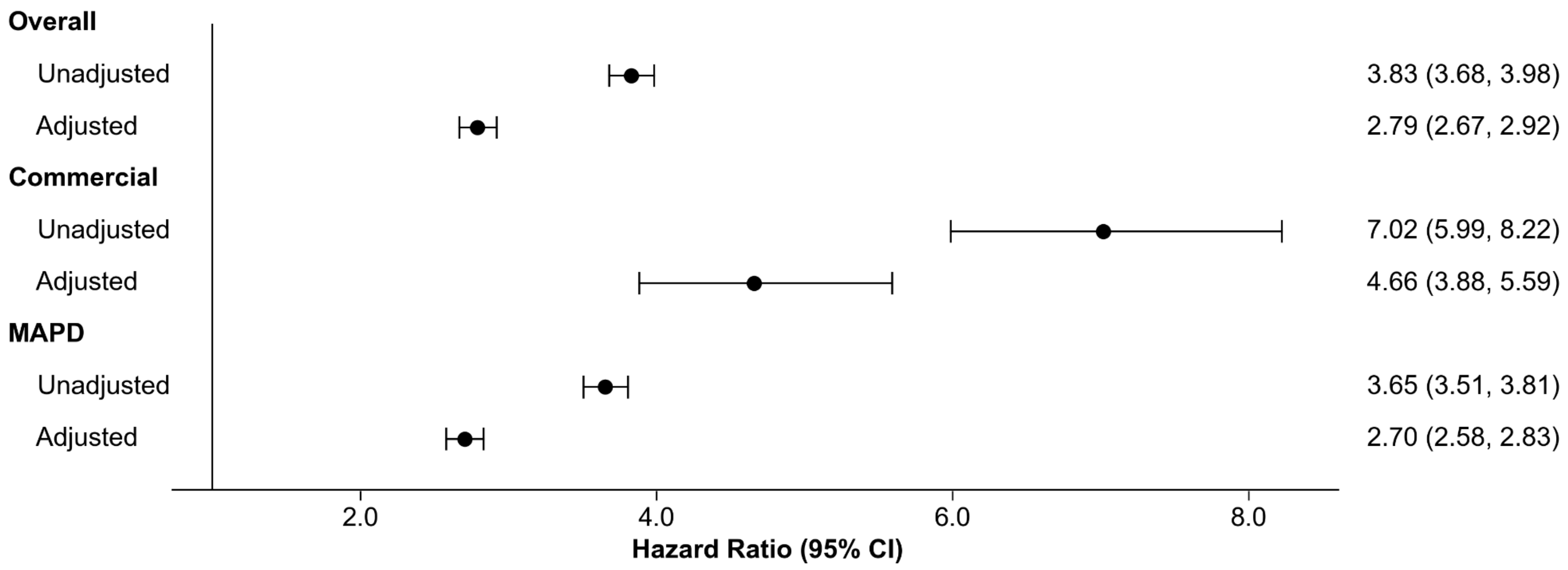

Abbreviations: CI, confidence interval; IPF, idiopathic pulmonary fibrosis.

Hospitalization were defined as respiratory-related if the claim had a diagnosis code for a respiratory condition (J00.xx-J99.xx) in the primary position or a procedure code for imaging testing (i.e., chest radiography, high-resolution CT [HRCT] chest).

Table S3. Proportional Hazards Model of Respiratory-Related Hospitalization with Clustering, Adjusted – Overall Population

Independent Variables	All-Cause Hospitalization			
	**Hazard Ratio**	**Lower 95% CI**	**Hazard Ratio**	***P***
Cohort				
Comparator	ref.	–	–	–
IPF	2.79	2.67	2.92	<0.001
Hospitalized during baseline	1.31	1.24	1.38	<0.001
Any medications filled during baseline	1.19	1.13	1.24	<0.001
**Other select comorbidities**				
Acute coronary syndrome (ACS)	1.16	1.10	1.22	<0.001
Angina	0.88	0.80	0.97	0.008
Atrial fibrillation	1.30	1.23	1.37	<0.001
Chronic kidney disease (CKD)	1.16	1.10	1.22	<0.001
Congestive heart failure (CHF)	1.44	1.36	1.52	<0.001
Depression	1.13	1.07	1.19	<0.001
Fatigue	1.12	1.07	1.17	<0.001
Gastroesophageal reflux disease (GERD)	1.07	1.02	1.11	0.007
Hypertension, arterial	1.22	1.16	1.29	<0.001
Lung cancer	1.41	1.18	1.68	<0.001
Myocardial infarction	1.12	1.04	1.20	0.004
Pulmonary embolism	1.03	0.89	1.19	0.702
Type 2 diabetes	1.24	1.18	1.29	<0.001
**AHRQ CCS comorbidities**				
Disorders of lipid metabolism	0.80	0.76	0.84	<0.001
Diseases of the heart	1.25	1.19	1.33	<0.001
Immunizations and screening for infectious disease	0.98	0.94	1.02	0.303
Diseases of the urinary system	1.11	1.06	1.17	<0.001
Other nutritional; endocrine; and metabolic disorders	0.96	0.92	1.00	0.038
Other connective tissue disease	1.08	1.03	1.13	<0.001
Diseases of arteries; arterioles; and capillaries	1.17	1.12	1.22	<0.001
Other nervous system disorders	1.12	1.07	1.18	<0.001
Spondylosis; intervertebral disc disorders; other back problems	1.01	0.97	1.06	0.570
Ear conditions	1.00	0.96	1.05	0.936

Observations read = 37,910, Observations used= 37,910.
Overall proportionality test <0.001.
Variable proportionality tests cohort_ana_r1 <0.001, B_IP 0.133, B_ACS <0.001, B_ANGINA 0.780, B_ATRFIB 0.029, B_CKD 0.291, B_CHF 0.567, B_DEPR 0.226, B_FATIGUE 0.770, B_GERD 0.577, B_HTSN 0.615, B_LUNGC 0.025, B_ACSMI 0.550, B_VTEPULEM 0.005, B_DIAB2 0.155, B_AHRQ_27 0.536, B_AHRQ_62 0.057, B_AHRQ_5 0.003, B_AHRQ_87 0.013, B_AHRQ_32 0.356, B_AHRQ_108 0.567, B_AHRQ_64 0.227.

Table S4. Generalized Linear Model With Gamma Distribution of All-Cause wPPPM Total Healthcare Costs, Adjusted – Overall Population

Independent Variables	All-cause Total Healthcare Costs (Medical + Pharmacy)				
	Cost Ratio	Lower 95% CI	Upper 95% CI	*P*	Predicted Value
**Cohort**					
Comparator	ref.	–	–	–	$1240
IPF	3.36	3.10	3.64	<0.001	$4163
Hospitalized during baseline	1.06	1.01	1.12	0.023	–
Any medications filled during baseline	1.39	1.33	1.46	<0.001	–
**Other select comorbidities**					
Acute coronary syndrome (ACS)	1.08	1.03	1.13	0.003	–
Angina	0.97	0.89	1.06	0.539	–
Atrial fibrillation	1.18	1.12	1.24	<0.001	–
Chronic kidney disease (CKD)	1.13	1.07	1.21	<0.001	–
Congestive heart failure (CHF)	1.15	1.09	1.22	<0.001	–
Depression	1.03	0.98	1.08	0.276	–
Fatigue	1.12	1.07	1.17	<0.001	–
Gastroesophageal reflux disease (GERD)	1.08	1.04	1.13	<0.001	–
Hypertension, arterial	1.02	0.96	1.07	0.598	–
Lung cancer	1.91	1.58	2.30	<0.001	–
Myocardial infarction	0.97	0.91	1.04	0.399	–
Pulmonary embolism	0.98	0.88	1.10	0.767	–
Type 2 diabetes	1.32	1.27	1.37	<0.001	–
**AHRQ CCS comorbidities**					
Disorders of lipid metabolism	0.94	0.89	0.99	0.010	–
Diseases of the heart	1.20	1.14	1.27	<0.001	–
Immunizations and screening for infectious disease	1.19	1.13	1.24	<0.001	–
Diseases of the urinary system	1.16	1.11	1.21	<0.001	–
Other nutritional; endocrine; and metabolic disorders	1.08	1.04	1.13	<0.001	–
Other connective tissue disease	1.08	1.04	1.13	<0.001	–
Diseases of arteries; arterioles; and capillaries	1.06	1.02	1.10	0.006	–
Other nervous system disorders	1.10	1.05	1.15	<0.001	–
Spondylosis; intervertebral disc disorders; other back problems	1.10	1.05	1.15	<0.001	–
Ear conditions	0.99	0.95	1.03	0.567	–

Observations read = 37,910, Observations used = 37,910.
Specification link test: p≤0.001.
Park test: estimate = 2.092, gamma distribution p-value = 0.653.
Park test p-value for Normal distribution: <0.001.
Park test p-value for Poisson distribution: <0.001.
Park test p-value for Gamma distribution: 0.653.
Park test p-value for Wald or Inverse Gaussian distribution: <0.001.
Weighted by the duration of observation time.

Figure S7. Respiratory-Related Costs

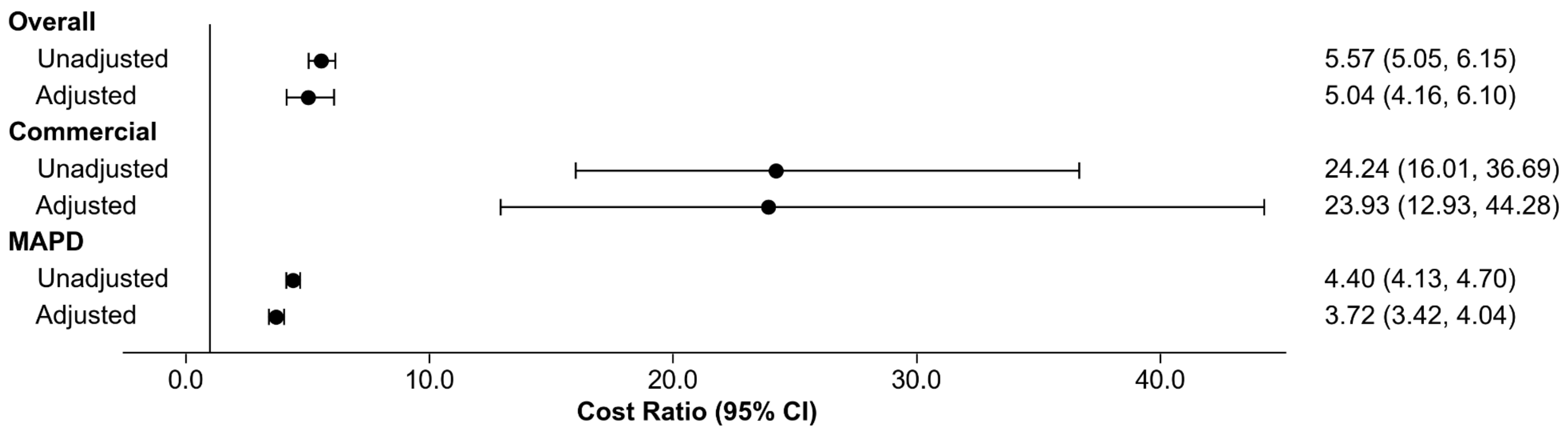

Abbreviations: CI, confidence interval; IPF, idiopathic pulmonary fibrosis.

Medical costs were defined as respiratory-related if the claim had a diagnosis code for a respiratory condition (J00.xx-J99.xx) in the primary position or a procedure code for imaging testing (ie. chest radiography, high-resolution CT [HRCT] chest).

Table S5. Generalized Linear Model With Gamma Distribution of Respiratory-Related wPPPM Total Medical Costs, Adjusted – Overall Population

Independent Variables	Respiratory-Related Healthcare Costs (Medical)				
	Cost Ratio	Lower 95% CI	Upper 95% CI	*P*	Predicted Value
**Cohort**					
Comparator	ref.	–	–	–	$314
IPF	5.04	4.16	6.10	<0.001	$1,580
Hospitalized during baseline	1.31	1.19	1.44	<0.001	–
Any medications filled during baseline	1.34	1.23	1.47	<0.001	–
**Other select comorbidities**					
Acute coronary syndrome (ACS)	1.23	1.12	1.34	<0.001	–
Angina	0.89	0.77	1.02	0.098	–
Atrial fibrillation	1.26	1.15	1.38	<0.001	–
Chronic kidney disease (CKD)	1.21	1.11	1.31	<0.001	–
Congestive heart failure (CHF)	1.41	1.27	1.57	<0.001	–
Depression	1.04	0.95	1.13	0.413	–
Fatigue	1.20	1.09	1.31	<0.001	–
Gastroesophageal reflux disease (GERD)	1.03	0.95	1.11	0.460	–
Hypertension, arterial	1.13	1.02	1.25	0.016	–
Lung cancer	2.14	1.65	2.77	<0.001	–
Myocardial infarction	1.01	0.91	1.13	0.829	–
Pulmonary embolism	0.91	0.73	1.13	0.379	–
Type 2 diabetes	1.33	1.24	1.43	<0.001	–
**AHRQ CCS comorbidities**					
Disorders of lipid metabolism	0.75	0.68	0.82	<0.001	–
Diseases of the heart	1.23	1.12	1.36	<0.001	–
Immunizations and screening for infectious disease	1.12	1.02	1.23	0.016	–
Diseases of the urinary system	1.04	0.97	1.13	0.257	–
Other nutritional; endocrine; and metabolic disorders	1.07	0.99	1.15	0.080	–
Other connective tissue disease	1.03	0.95	1.11	0.522	–
Diseases of arteries; arterioles; and capillaries	1.15	1.07	1.24	<0.001	–
Other nervous system disorders	1.09	1.01	1.18	0.036	–
Spondylosis; intervertebral disc disorders; other back problems	1.07	0.98	1.16	0.118	–
Ear conditions	0.93	0.87	1.01	0.080	–

Observations read = 37,910, Observations used = 37,910.
Specification link test: p≤0.001.
Park test: estimate = 2.300, gamma distribution p-value = 0.245.
Park test p-value for Normal distribution: <0.001.
Park test p-value for Poisson distribution: <0.001.
Park test p-value for Gamma distribution: 0.245.
Park test p-value for Wald or Inverse Gaussian distribution: 0.007.
Weighted by the duration of observation time.
